# Small molecule inhibiting microglial nitric oxide release could become a potential treatment for neuroinflammation

**DOI:** 10.1371/journal.pone.0278325

**Published:** 2023-02-06

**Authors:** Philipp Jordan, Amanda Costa, Edgar Specker, Oliver Popp, Andrea Volkamer, Regina Piske, Tessa Obrusnik, Sabrina Kleissle, Kevin Stuke, Andre Rex, Martin Neuenschwander, Jens Peter von Kries, Marc Nazare, Phillip Mertins, Helmut Kettenmann, Susanne A. Wolf

**Affiliations:** 1 Max-Delbrück-Center for Molecular Medicine, Cellular Neuroscience, Berlin, Germany; 2 Department of Neurology, Washington University School of Medicine, St. Louis, MO, United States of America; 3 Screening Unit, Leibniz Research Institute for Molecular Pharmacology FMP, Berlin, Germany; 4 Medicinal Chemistry Research Group, Leibniz Research Institute for Molecular Pharmacology FMP, Berlin, Germany; 5 Max-Delbrück-Center for Molecular Medicine, Mass spectroscopy and Proteomics, Berlin, Germany; 6 Dept. of Physiology, In Silico Toxicology and Structural Bioinformatics, Charité-Universitätsmedizin Berlin, Berlin, Germany; 7 Max Delbrück Center for Molecular Medicine in the Helmholtz Association, RNAi Platform, Berlin, Germany; 8 Centre for Stroke Research, Charité-Universitätsmedizin Berlin, Corporate member of Freie Universität Berlin, Humboldt-Universität zu Berlin, and Berlin Institute of Health, Berlin, Germany; 9 Shenzhen Institute of Advanced Technology, Chinese Academy of Sciences, Shenzhen, China; 10 Dept. Ophthalmology, Charité-Universitätsmedizin Berlin, Berlin, Germany; Albany Medical College, UNITED STATES

## Abstract

Microglia are the immune effector cells of the central nervous system (CNS) and react to pathologic events with a complex process including the release of nitric oxide (NO). NO is a free radical, which is toxic for all cells at high concentrations. To target an exaggerated NO release, we tested a library of 16 544 chemical compounds for their effect on lipopolysaccharide (LPS)-induced NO release in cell line and primary neonatal microglia. We identified a compound (C1) which significantly reduced NO release in a dose-dependent manner, with a low IC_50_ (252 nM) and no toxic side effects *in vitro* or *in vivo*. Target finding strategies such as *in silico* modelling and mass spectroscopy hint towards a direct interaction between C1 and the nitric oxide synthase making C1 a great candidate for specific intra-cellular interaction with the NO producing machinery.

## Introduction

Microglia are immune cells of the central nervous system (CNS) and upon an insult release pro-inflammatory molecules such as tumour necrosis factor α (TNFα) [1], Interleukin 1 β (IL1β) [2], Interleukin 6 (IL6) [3], and nitric oxide (NO) [4, 5]. Microglia are professional phagocytes of the brain [6] and migrate towards injury [7, 8]. A simplified paradigm of microglia activation uses lipopolysaccharide (LPS), a bacterial component, which triggers a well-established pro-inflammatory response, resulting in the release of NO. In high intracellular concentrations, NO reacts non-specifically with proteins, nucleic acids and lipids, affecting the host and invader to the same extent. In this sense, even though NO release potentiates inflammation, it also induces damage of the host tissue [9].

NO is produced by the nitric oxide synthase (NOS), an enzyme that catalyses the conversion of L-arginine to citrulline and NO. In humans and mice, three isoforms of NOS are known: eNOS, nNOS, and iNOS. eNOS and nNOS are constitutively expressed by endothelial cells and neurons, respectively, and are regulated by cytosolic calcium [10]. iNOS is induced by a pathologic event in microglia and macrophages, in a calcium independent manner [11]. NO plays a role in a plethora of diseases [5], such as diabetes [12], hypertension [13], cancer [14], drug addiction [15], stroke [16], intestinal motility disorders [17], memory and learning disorders [18, 19], septic shock [20], inflammatory and autoimmune diseases [21]. Since excessive NO can damage tissue and organs, a therapeutic relevant antagonizing compound could potentially ameliorate secondary damage in the above-mentioned diseases. Others have tried to target NO release in different diseases with limited or null success. The general NOS inhibitor L-NMMA raises cardiovascular safety concerns and has an unfavourable pharmacokinetic profile [22]. Therefore, a specific iNOS inhibitor would be advantageous over general NO inhibitors. Currently some specific iNOS inhibitors are known with difference in activity and selectivity [23, 24]. For instance, Aminoguanidine is a highly reactive nucleophilic reagent that interacts with many biological molecules (pyridoxal phosphate, pyruvate, glucose, malondialdehyde, and others) [25]. A clinical trial using aminoguanidine to prevent progression of diabetic nephropathy was terminated early due to safety concerns and apparent lack of efficacy [26]. Other candidates that were tested in the clinical setting like GW274150 and GW273629 are potent and highly selective inhibitors of iNOS *in vitro* and *in vivo*. However, GW273629 has an unfavourable pharmacokinetic profile, and GW274150 was used in phase I/II clinical trials for arthritis, migraine, and asthma with insignificant or not published results [27]. Some promising results by selective iNOS inhibitors of microglia (BV2 cells) were recently reported [28], suggesting the potential usefulness of iNOS inhibitors in redirecting microglia from detrimental to pro-regenerative phenotype.

We here analysed a library of 16 544 drug-like compounds to identify an inhibitor of mNO production in microglia using a cell line and primary cells. We used qPCR, *in silico* modelling and mass spectroscopy and uncovered nitric oxide as a potential target for C1. We further tested the compound’s bioavailability *in vivo* and its effect in a mouse model for mild middle cerebral occlusion (MCAo).

## Methods

### Solutions

If not stated otherwise, all cells were incubated in Dulbecco’s Modified Eagle Medium (DMEM) (GIBCO, Thermo Fisher Scientific, and Waltham, USA), containing 10% foetal calf serum (FCS) and Penicillin-Streptomycin-Glutamine (Thermo Fisher Scientific), in this manuscript referred to as “plain medium”.

### Primary cultured neonatal microglia

For preparation of cultured neonatal microglia, P0–P3 C57BL/6 mice were used. After discarding the meninges and cerebellum 10 brains were collected in tubes containing Hank’s balanced salt solution (HBSS) on ice. The dissected brains were washed 3 times before adding 400 μL trypsin (0.1 mg/L) and DNase (5 μg/L) in phosphate buffered saline (PBS). After 2 min of incubation with this solution, the reaction was blocked by adding plain medium. The medium was discarded, 1 mg DNase was added, and the cells were mechanically dissociated with a Pasteur pipette followed by a glass pipette. The cells were centrifuged for 10 min at 129 g at 4°C. The supernatant was discarded. The pellet was resuspended in plain medium and plated (2.5 brains/flask) in Poly-L-Lysine coated flasks. The cells were washed after 2 days with PBS and incubated for another 7 days in plain medium. Afterwards the medium was replaced by a plain medium with 33% L929 conditioned medium. After 2 days, cells were harvested by shaking them for 30 min at 150 rpm. The supernatant was centrifuged for 10 min at 129g at 4°C. The cells were plated into a 96 well plate (100 000 cells/well, 200 μL medium per well). This procedure could be repeated twice with a 2-day interval.

### Primary cultured neonatal astrocytes

The preparation of primary cultured neonatal astrocytes followed the previously described preparation of the primary cultured neonatal microglia. After the third shake off microglia, the remaining astrocytes were trypsinized, washed with PBS and seeded into a 96 well plate (10 000 cells/well, 200 μL medium per well).

### Primary cultured macrophages

Bone marrow macrophages were isolated from C57BL/6 adult mice (P49-56) as previously described [29]. In summary, mice were killed by cervical dislocation and the femurs were removed by cutting through the tibia near the pelvic bone and below the knee. Any muscle connected to the bone was carefully removed. Ice-cold sterile PBS was slowly flushed through the bone and the contents were collected in a sterile 15 mL polypropylene tube on ice. After centrifugation for 10 min at 200 g at 4°C, red blood cells were lysed by adding 3–10 mL ammonium chloride solution. After a 3–5 min recovery period, the suspension was centrifuged for 10 min at 200 g at 4°C and the supernatant was removed. Cells were plated in a 10 cm dish with plain medium containing 10 ng/mL M-CSF and cultured for 7 days to allow differentiation. We plated 50 000 cells with 200 μL medium per well in a 96 well plate.

### Cell culture of immortalized cell lines: Microglia cell line BV-2, and oligodendrocyte cell line OLN-93

The immortalized murine microglia cell line BV-2 [30] and the oligodendrocyte cell line OLN-93 [31] were incubated in T75 flasks with plain medium. The cells were split every 2 to 3 days at the dilution of 1:10 before they reached confluence. To do so, the cells were washed twice with PBS, followed by incubation of up to 5 min with trypsin. The trypsinization was stopped using plain medium. The cells were centrifuged at 300 g for 10 min at 4°C, the supernatant was discarded, and the cells were resuspended in a new medium and seeded into a new flask.

### Setup of the compound library

A library of 16 544 compounds (ChemBioNet library) was tested at the Screening Unit core facility of the Leibniz-Forschungsinstitut für Molekulare Pharmakologie. The library consists of a diverse set of compounds that was designed on the basis of the maximum-common substructure principle [32]. The compounds of the screening library are arranged on 384-well microtiter plates, in which compounds are placed into columns 1–22 as 10 mM solutions dissolved in dimethyl sulfoxide (DMSO). DMSO alone is placed into columns 23 and 24, therefore permitting to screen 352 compounds per plate together with 32 controls.

### High throughput screening (HTS)

Using a dispenser (EL406, Biotek, Winooski, USA), BV-2 cells were seeded at a cell density of 5 000 cells / 40 μL per well into a 384-well plate (3683, Corning, New York, USA). The plates were incubated for 24 hours at 37°C, 5% CO_2_ and 95% humidity. Using a robotic liquid-handler (Freedom Evo, Tecan, and Maennedorf, Switzerland), compounds were pre-diluted in cell medium to 500 μM and 500 nl were added into each well. After 1-hour pre-incubation of the compounds with the cells, 10 μL of 5 μg/mL LPS solution was added to columns 1–23 using a dispenser. The final assay conditions were therefore 50-μL total volume containing 5 μM of test compound, 0.05% DMSO, and 1 μg/mL LPS. The positive controls were treated with LPS only in column 23 and negative controls were left in plain medium in column 24. After 48 hours of incubation with LPS and compounds, 25 μL of 2-fold concentrated Griess reagent was added and absorbance was read at 540 nm in a plate reader (Safire2, Tecan, and Maennedorf, Switzerland). Active compounds were identified by observing decreased Z-scores of the absorbance signal.

### Experimental design and statistical analysis for HTS

Data was normalized for each plate by using statistically robust estimators [33].


$$Z-score=\frac{x_{i}-Median}{MAD*1.48258}$$ 1)

*Z-score* indicates how many standard deviations an observation is above or below the median (1). *x*_*i*_ is the signal of a single sample; *Median* denotes the median signal on a plate without the controls, and MAD the median absolute deviation on a plate without the controls.


$$Percent\;Activity=\frac{x_{i}-Neg}{|pos-Neg|}$$ 2)

*Percent activity* measures the response relative to an unperturbed state (2). *Neg* is the median of the negative (no LPS induction) control samples, and *Pos* is the median of the positive (= 100% LPS-induced, non-treated) control samples on a plate.


$$Z^{\prime }=1-\frac{3*(\delta^{P}+\sigma^{N})}{\left|\mu^{P}-\mu^{N}\right|}$$ 3)

*Z’* is a common statistical tool used to measure the effective dynamic signal range of HTS assays (3) and serves as a quality control metric. *δ*_*p*_ and *δ*_*n*_ are the standard deviations of the positive and negative controls, respectively, and *μ*_*p*_ and *μ*_*n*_ are the mean values of the positive and negative controls of a plate.


f(x)=c+(d−c)/(1+exp(b*(log(x)−log(e))) 4)

IC_50_ determination was carried out using the four-parameter log-logistic function (4), and the Pipeline Pilot curve fit module for determining dose-response curves using ILRS algorithm. *b*: Hill-coefficient (steepness of the IC_50_ curve at the inflection point), e: IC_50_ value, *c* and d: left and right activity asymptotes.

Data was pre-processed (initial graphical quality control and data normalization) using in-house software, reports containing chemical structures were generated using Pipeline Pilot (Biovia).

### Concentration-dependent validation and viability determination in primary murine microglia

Concentrated stock solutions of compounds identified from primary screening were rearranged onto a new 384-well plate, and 10 sequential 2-fold serial dilutions were created across multiple plates in DMSO. Starting from these diluted compound mother plates, the protocol was repeated exactly as for primary screening to span the concentration range between 19.5 nM and 20 μM. In order to obtain the 10 and 20 μM concentration points, 1000 nl and 2000 nl were transferred from the 500 μM pre-dilution plate, respectively. Each concentration was measured in duplicates. Normalized percent activities were plotted against the compound concentration to obtain the IC_50_ values.

To obtain information about the impact of compounds on cell viability, a second batch of plates was prepared as described above, but 5 μl of Alamar Blue solution was added after 48 hours instead of Griess reagent and incubation was continued for additional 4 hours at 37°C. Finally, the resorufin fluorescence was detected at an excitation wavelength of 530 nm and emission of 570 nm. Since no control samples were present on the assay plate for data normalization of cell viability, the raw data values were added on the second y-scale to the IC_50_ plots.

### Griess assay

Cells were seeded according to the protocols described above, after compounds and stimuli were added, 100 μL of supernatant was transferred into a new 96 well plate and 100 μL freshly mixed Griess reagent were added. Griess reagent was composed of reagent A (100 mg Naphthylethylene in 50 mL aqua dest.) and reagent B (1 g Sulfanilamide, 6 ml H3PO4 (85%) in 44 mL aqua dest.) mixed 1:1. The solution was carefully mixed, and the absorbance was determined in a microplate reader at a wavelength of 550 nm. Dissolved sodium nitrite in plain medium served as a standard. Total amount of nitrite was calculated by a linear regression of the standard curve.

### AlamarBlue assay

Cells were seeded according to the protocols described above, after compounds and stimuli were added, the cells were washed once with HBSS (37°C) and 100 μL of a 1:10 AlamarBlue (Thermo Fisher Scientific) dilution in plain medium was added. The conversion of resazurin to resorufin was measured by absorbance (absorbance wavelength of 570 nm and a reference wavelength of 600 nm) after 3 hours.

### BCA-assay

The BCA-assay was performed according to the manufacturer’s protocol. In brief, 5 μL of cell lysate was diluted in 45 μL H_2_O. 5 μL of this dilution was stocked with 100-μL BCA-Kit mixture (50:1, A:B). After 30 minutes of incubation shaking at 37°C the absorption at 550 nm was measured using an Infinite M200 multi detection plate reader (TECAN). The protein concentration was calculated using Albumin (BSA) Standards (Thermo Fisher Scientific).

### Enzyme-linked immunosorbent assay (ELISA)

Cells were seeded according to the protocols described above, after compounds and stimuli were added, the supernatant was collected, centrifuged (500 g, 5 minutes) and stored at -20°C until analysed. The following kits from Bio-Techne (Minneapolis, USA) were used to detect IL1β, IL6 and TNFα: ELISA Mouse IL-1β/IL-1F2 DuoSet, ELISA Mouse IL-6, and ELISA Mouse TNFα. We performed the ELISA assays according to the manufacturer’s protocol.

### Micro chemotaxis assay

The effect of the compound on directed migration and general motility was tested using a 48-well micro chemotaxis Boyden chamber (Neuroprobe, Gaithersburg, USA). Upper and lower wells were separated by polycarbonate filters (8 μm pore size; Neuroprobe, Gaithersburg, USA). Microglial cells (2–4 × 10^4^ cells) and 50-μL plain medium were added to the upper compartment. In addition, 2.5 μM of compound in DMSO, 125x10^-5^ v/v DMSO respectively, and/or 100 μM ATP were added to the upper and/or lower chamber, as shown in **Fig 3A and 3B.** Plain medium was used as a control. The chamber was incubated at 37°C and 5% CO_2_ for 6 h. Cells remaining on the upper surface of the membrane were removed by wiping, and cells in the lower compartment were fixed in methanol for 10 min and subjected to Diff-Quik stain (Medion Grifols Diagnostics AG, Düdingen, Switzerland). The rate of microglial migration was calculated by counting cells in four random fields of each well using a 20× bright-field objective. All data were normalized to the ATP-induced chemotaxis.

### Flow cytometry-based phagocytosis assay

10^6^ primary cultured microglial cells were seeded into 3.5 cm dishes in plain medium overnight at standard conditions. The cells were treated for 1 hour with 2.5 μM C1 in DMSO, 125x10^-5^ v/v DMSO respectively, or plain medium only. To stimulate microglia, LPS (1 μg/mL) was added for an additional 24 hours. Fluoresbrite Carboxylate Microspheres (BrightBlue, 4.5 μm, Polyscience, Niles, USA) were coated with foetal calf serum for 30 min at room temperature, and subsequently centrifuged at 3000 g for 2 min at room temperature. The beads were resuspended in HBSS at a final concentration of 2×10^6^ beads/ mL. The cells were washed once with HBSS (37°C) and incubated with 1 mL bead solution for 30 min at 37°C. Afterwards, microglia were washed twice with ice cold HBSS, scratched off and pulled down at 500 g for 5 min. Dead cells were stained with a propidium iodide solution (1:200 in HBSS). The median intensity of the bright blue beads was measured using a BD LSRFortessa Flow cytometer (BD Bioscience, Sparks, USA), and calculated using FlowJo v10 software (Ashland, USA). The data of each experiment was normalized to the unstimulated media control.

### Quantitative PCR

The same stimulation protocol as for the Flow cytometry-based phagocytosis assay was applied. Cells were seeded overnight, treated with 2.5 μM C1 in DMSO, 125x10^-5^ v/v DMSO respectively, or plain medium only for 1 hour, followed by an additional stimulation with 1 μg/mL LPS for 24 hours. Total RNA was isolated using the RNeasy Plus Mini Kit (Qiagen, Hilden, Germany). On-column DNase 1 (Qiagen) digestion was performed, and total RNA was eluted in RNase-free water. RNA yield was measured using a Nanodrop 1000 (Thermo Fisher Scientific) spectrophotometer and quality was assessed using an Agilent 2100 Bioanalyzer (Agilent, Santa Clara, USA). Samples were stored at -80°C until further use. First-strand cDNA synthesis was performed with the SuperScript II reverse transcriptase (Thermo Fisher Scientific) using oligo-dT primers 12–18 (Invitrogen) according to the manufacturer’s instructions. Quantitative real-time PCR (qRT PCR) reactions were performed in a 7500 Fast Real-Time thermocycler (Thermo Fisher Scientific) using the SYBR Select Master Mix (Thermo Fisher Scientific) according to the manufacturer’s instructions. cDNA input ranged between 1 and 5 ng/μl of total RNA transcribed into cDNA. The results were normalized to the expression of βActin of the same sample. Primers used are following: iNOS forward TCACGCTTGGGTCTTGTTCA, iNOS reverse TGAAGAGAAACTTCCAGGGGC, βActin forward CGTGGGCCGCCCTAGGCACCA, and βActin reverse CTTAGGGTTCAGGGGGGC.

### Propidium iodide-based proliferation and cell death assay

To determine the relative proliferation and cell death, the cells were seeded into a 96 well plate, let adhere for 24 hours and afterwards treated for additional 48 hours. The supernatant was removed, and the cells were washed carefully with 37°C HBSS once. 1:200 propidium iodide PBS solution was added, and the cells were incubated for 10 minutes. The intensity of the propidium iodide signal of the dead cells was measured using a microplate reader. Afterwards, all cells were killed with a 10 min incubation of 10% DMSO and the propidium iodide signal of all cells was measured. All signals were corrected for the background noise, subtracting the blank signal. The percentage of dead cells was calculated by dividing the dead-cell-signal by the all-cell-signal. Proliferation was calculated as a relative value to the plain medium control.

### Intravenous pharmacokinetic study in mice

The company Touchstone Biosciences (Plymouth Meeting, USA), according to their standard procedures carried out the intravenous pharmacokinetic study in mice. In brief, three male adult mice of the CD-1 strain were fasted overnight. 5 mg/kg compound was given intravenously in one shot. After 5, 15, 30 min, and 1, 2, 4-, 6-, 8-, and 24-hours blood samples were collected from the vein and analysed via Liquid chromatography-mass spectrometry. After 24 hours, all mice were killed.

The same company carried out the intravenous tissue distribution study in mice. In brief, three male adult mice of the strain CD-1 per time point (4 time points) were fasted overnight. 5 mg/kg compound was given intravenously. After 30 min, 1, 2, or 4 hours the blood, brain, heart, liver and kidney were extracted and the level of compound concentration for each organ and the blood were calculated using liquid chromatography-mass spectrometry.

### Animals and group allocation for middle cerebral artery occlusion (MCAo) study

C57BL/6 (13 weeks old, Charles River, Germany) male mice were handled according to governmental and internal rules and regulations, having free access to food and water. The experiments were approved by the local animal welfare committee (LaGeSo) under the license number GO—249/15. A total of 24 male mice were analysed after undergoing 30 minutes of left sided MCAo. Animals were randomly attributed to treatment paradigms, and experimenters were blinded at all stages of interventions. The mice were injected intraperitoneal (i.p.) every day for 7 days, after the behavioural tests were performed, with the compound (n = 11) or with the vehicle (125x10^-5^ v/v DMSO = 0.00125% DMSO, n = 13).

### Experimental design and statistical analysis

To determine the number of mice in each group, we used previous experimental data and G power analysis. Based on previous experiments, in order to detect a difference in the lesion size with 90% power, using the GPower® software (Heinrich Heine University of Duesseldorf, Germany) [34, 35], we determined to need a minimum of 11 mice in each group. We considered a 30% drop out rate due to the experimental procedure. We started with a number of 36 mice: 6 mice (4 treated and 2 controls) displayed no lesion on MRT scans and were therefore excluded from the study, 2 mice died during the MCAo procedure, and 2 mice died on day 4 after MCAo (one in each group). One mouse in each group was excluded from the study since their ischemic volume was considered a significant outlier within the group, using the Grubbs’ test. Thus, 24 out of 36 mice were included into the analysis (34% drop out rate). We have then performed the experiments with 11 mice in the treatment group and 13 in the control group.

Statistical evaluation was performed using PRISM version 5.0 software (Graph Pad, La Jolla, USA). Data from experiments using animals were analysed using planned comparisons to test the following questions of primary interest 1) is the compound able to improve stroke related motor impairment and 2) does the compound have an effect on the ischemic lesion volume. Comparisons between multiple experimental groups were made using one or two-way ANOVA with Bonferroni’s post-hoc test when appropriate. For comparisons between a single experimental group and a control group, we used Student’s t test. p<0.05 was considered to be statistically significant. Data are given as mean ± SEM. Details on statistical analyses and experimental design, including which tests were performed, exact p-values (within resolution of software limits), sample sizes (n), and replicates, are provided within the legend of each figure.

### Induction of cerebral ischemia

Mice were anesthetized with 3–4% isoflurane and maintained in 1.5% isoflurane in 70% N_2_O and 30% O_2_ using a vaporizer. MCAo was essentially performed as described elsewhere [36], http://dx.doi.org/10.17169/refubium-1690). In brief, brain ischemia was induced with a silicone rubber-coated monofilament 7–0, diameter 0.06–0.09 mm, length 20 mm; diameter with coating 0.19 ±0.01 mm; coating length 9–10 mm. The filament was introduced into the internal carotid artery up to the anterior cerebral artery. Thereby, the middle cerebral artery and anterior choroidal arteries were occluded. The filament was removed after 30 min to allow reperfusion.

### Determination of infarct volume

#### Magnetic resonance imaging

MRI was performed using a 7 Tesla rodent scanner (Pharmascan 70 ⁄ 16, Bruker BioSpin, Bruker, Billerica, USA) with a 16 cm horizontal bore magnet and a 9 cm (inner diameter) shielded gradient with an H-resonance-frequency of 300 MHz and a maximum gradient strength of 300 mT/m. For imaging a 20mm - 1H-RF quadrature-volume resonator with an inner diameter of 20 mm was used. Data acquisition and image processing were carried out with the Bruker software Paravision 5.1.

During the examinations mice were placed on a heated circulating water blanket to ensure constant body temperature of 37°C. Anesthesia was induced with 2.5% and maintained with 2.0–1.5% isoflurane (Forene, Abbot, Wiesbaden, Germany) delivered in an O_2_ / N_2_ mixture (0.3 / 0.7 L/min) via a facemask under constant ventilation monitoring (Small Animal Monitoring & Gating System, SA Instruments, New York, USA).

For imaging the mouse brain, a T2-weighted 2D turbo spin-echo sequence was used (imaging parameters TR / TE = 4200 / 36 ms, rare factor 8, 4 averages, 32 axial slices with a slice thickness of 0.5 mm, field of view of 2.56 x 2.56 cm, matrix size 256 x 256).

#### Image analysis

Calculation of lesion volume was carried out with the program Analyze 10.0 (AnalyzeDirect, Inc., Overland Park, USA). The hyper intense ischemic areas in axial T2-weighted images were assigned with a region of interest tool. This enables threshold-based segmentation by connecting all pixels within a specified threshold range about the selected seed pixel and results in a 3D object map of the whole stroke region. Further, the total volume of the whole object map was automatically calculated.

### Motor deficits assessment

#### Accelerated rotarod test

The Rotarod test was performed to access motor coordination using a treadmill with a diameter of 3 cm (TSE Systems, Chesterfield, USA). This test was performed with accelerating velocity (4–40 rpm) and maximal velocity was achieved after 300 s. The time until the animals dropped was measured. Animals were trained on day 2 and 3 before MCAo and baseline was taken on the day before MCAo. Tests were always performed four times and daily means were used for statistical analysis.

#### Pole test

A vertical pole (80 cm high with rough surface) was used for this test, to analyse extrapyramidal motor locomotion. Mice were placed head upwards on the top of the pole. The time taken to orientate the body completely downwards, making a 180° turn, and to reach the floor with all four paws were recorded. If the animal was unsuccessful for either task, it was scored the maximum time that any other animal from the same experimental group took to perform the task. Animals were trained on day 2 and 3 before MCAo and baseline was taken on the day before MCAo. Tests were always performed after the accelerated Rotarod test and repeated four times and means were used for statistical analysis.

#### Corner test

Each mouse was placed on a cage containing two vertical boards attached to each other forming an angle of 30° in 2 of the corners. The side chosen to leave the corner once it made contact to the boards with its whiskers was observed within 10 trials per day. Whereas healthy animals leave the corner without side preference, mice after stroke preferentially leave the corner towards the non-impaired (i.e., left) body side [37]. Baseline side preference was accessed on day 5 before MCAo and the mice were tested again on day 6 after MCAo.

### *In silico* modelling: Protein-ligand docking

#### Structure selection

The human iNOS PDB structure 3e7g [38] was chosen due to the good (low) resolution of 2.2 Å and the co-crystallization with inhibitor AR-C95791 (PDB identifier: AT2). Correct protonation states were calculated with Protoss [39].

#### Docking

To generate docking poses for the compounds C1-*S* and C1-*R*, LeadIT v.2.1.9 suite from BioSolveIT (www.biosolveit.de/LeadIT Sankt Augustin, Germany 2017, version 2.3.3) was used (docking algorithm FlexX) [40]. The protein was prepared as follows: Chain A and B from PDB 3e7g were kept; the cofactor heme as well as metal ions were annotated as part of the protein; the cofactor H4B was excluded. The binding site was defined including all residues within 8Å around the co-crystallized ligand from chain A, AT2_A. First, AT2_A was re-docked, and second the two compounds C1-*S* and C1-*R* were docked, generating 50 poses per compound.

#### Rescoring

To optimize and rescore the docking poses, SeeSAR v.8.0 from BioSolveIT (www.biosolveit.de/SeeSARk Sankt Augustin, Germany 2020) was used. The same protonated 3e7g structure was used (chain A only, including heme and Fe). The binding site was defined using the co-crystallized ligand. In SeeSAR, the best docking poses per compound from LeadIT were used as starting structures. 10 new poses were generated per compound and evaluated with the built-in HYDE scoring function [41]. The poses with the best estimated affinities were chosen for further analysis. Moreover, iNOS revealed a better estimated binding affinity to C1-S (nano-molar) than the two other NOS species (estimated affinity micro-molar).

#### Visualization

PyMOL v.2.3.0. was used to further analyse and visualize the predicted binding poses, as well as to create the 3D images [42].

### Mass spectrometry

#### Cell preparation

10^6^ BV-2 cells were plated on TC100 cell culture dishes in 20 mL DMEM complete and let rest overnight. 10 mL medium were replaced with medium containing 10μM C1-*S*, C1-*R*, or without compound. After 1 hour, the cells were either stimulated with 1 μg/mL LPS or left unstimulated. After 24h incubation time, the cells were scraped, centrifuged at 500g for 5 min at 4°C, and washed with 10 mL PBS once. The pellet was transferred to a protein-free Eppendorf tube in 1 mL PBS, centrifuged at 300g for 5 min at 4°C, the remaining pellet was snap-frozen on dry ice and stored at -80°C until further usage.

### Protein immunoprecipitation

For label-free proteomics analysis, samples were subjected to tryptic on-bead digest [40]. The cell pellets were lysed in 150 μL lysis buffer. After a 20 min rest on ice, the samples were centrifuged at 14 000g for 10 min and 4°C to remove the cell debris. A BCA was done with the remaining supernatants to determine the protein concentration. The sample with the lowest protein concentration was the reference sample, and all other samples were diluted with a lysis buffer with protease and phosphatase inhibitor to ensure that all samples had the same protein concentration. Streptavidin beads were washed 3 times with lysis buffer without protease and phosphatase inhibitors to clean them and then diluted in the same volume of lysis buffer as they were originally stored in. 100 μL of the bead solution was added to 150 μL of each lysate and incubated overnight at 4°C, allowing the Streptavidin beads to bind to the biotin-tag of the compounds and therefore the coupling of proteins. Based on the fact that streptavidin beads are magnetic, the beads and the coupled compound with proteins could be fished out by placing the Eppendorf tubes on a magnetic rack and removing the supernatant. With the same technique the beads were washed 5 times with 1 mL washing buffer. To elute the proteins from the beads, the remaining bead pellets were incubated with an 80 μL digestion buffer, consisting of trypsin at 25°C on a shaker at 1000 rpm for 1h. The magnetic beads were separated from the liquid supernatant by placing the Eppendorf tubes on a magnetic rack and transferring the remaining bead-free liquid to a new Eppendorf tube. The beads were washed twice with 60 μL washing buffer without trypsin or DTT, and the supernatants of all washes from each sample were combined. To reduce the eluted liquids, 4 mM DTT was added. The samples were then incubated at 25°C on a shaker at 1000 rpm for 30 min. The reduced proteins were alkylated by incubating the samples with 10 mM Iodoacetamide protected from light on a shaker with the previous settings. After adding 1 μg of trypsin per sample, they were placed in a shaker protected from light at 25°C and 700 rpm overnight. To clean-up the digestion, the samples were acidified by adding 1% form aldehyde. To store the samples safely, stage-tipping was performed. Therefore 5 steps were done, each followed by a 3000g spin-down. C18 membrane disks were activated by 50 μL MeOH. After the centrifugation, the stage-tips were conditioned with 100 μL of 50% ACN/0,1% FA followed by two treatments with 100 μL of 3% ACN/0,1% FA. To each stage-tip, a single sample was added and centrifuged. The membranes were then washed with 100 μL of 3% ACN/0,1% FA twice, and the bound peptides were eluted with 50 μL of 50%MeCN/0,1% FA. The liquid samples containing the peptides were then transferred to the measuring plate. By usage of a speed-vac, the peptides were lyophilized and snap-frozen. The samples were stored at -20°C until measured in the mass spectrometer.

#### Sample preparation and analysis

LC-MS acquisition occurred on an orbitrap Exploris 480 mass spectrometer (Thermo Fisher Scientific) in data-dependent MS2-mode. Database search was done with MaxQuant (version 1.6.3.1 [43] using an FDR of 0.01 for peptides as well as proteins against a Uniprot mouse database (July 2018). MS intensities were normalized by the MaxLFQ algorithm [44] while using the match-between-runs feature within each compound group. Further data analysis was done using R. A requirement of at least three valid values in one of the groups to be compared was set before applying column-wise imputation using a randomized Gaussian distribution with a width of 0.2 and a downshift of 1.8. Values among the replicates of the groups to be related were used for comparison applying a two-sample moderated t-test (limma R package; [45]). Significance calling on proteins was done after multiple comparison correction by calculating adjusted p-values (q values) with the Benjamini-Hochberg method.

### Statistical analysis

Massspectroscopy and HTS-screening statistics have been described in the respective methods sections.

Depending on the number of groups and variables compared, we either used a 1 way ANOVA followed by a Bonferroni post hoc test or a 2 way ANOVA followed by Sidak post hoc test. The respective tests used are indicated in the figure legends. For convenience, we prepared a S1 Table containing all the information regarding statistical test and sample size for each single experiment. Raw data are stored in the Dryad repository DOI https://doi.org/10.5061/dryad.q83bk3jms.

## Results

### Screening for compounds inhibiting nitric oxide release in microglia

**Fig 1:**
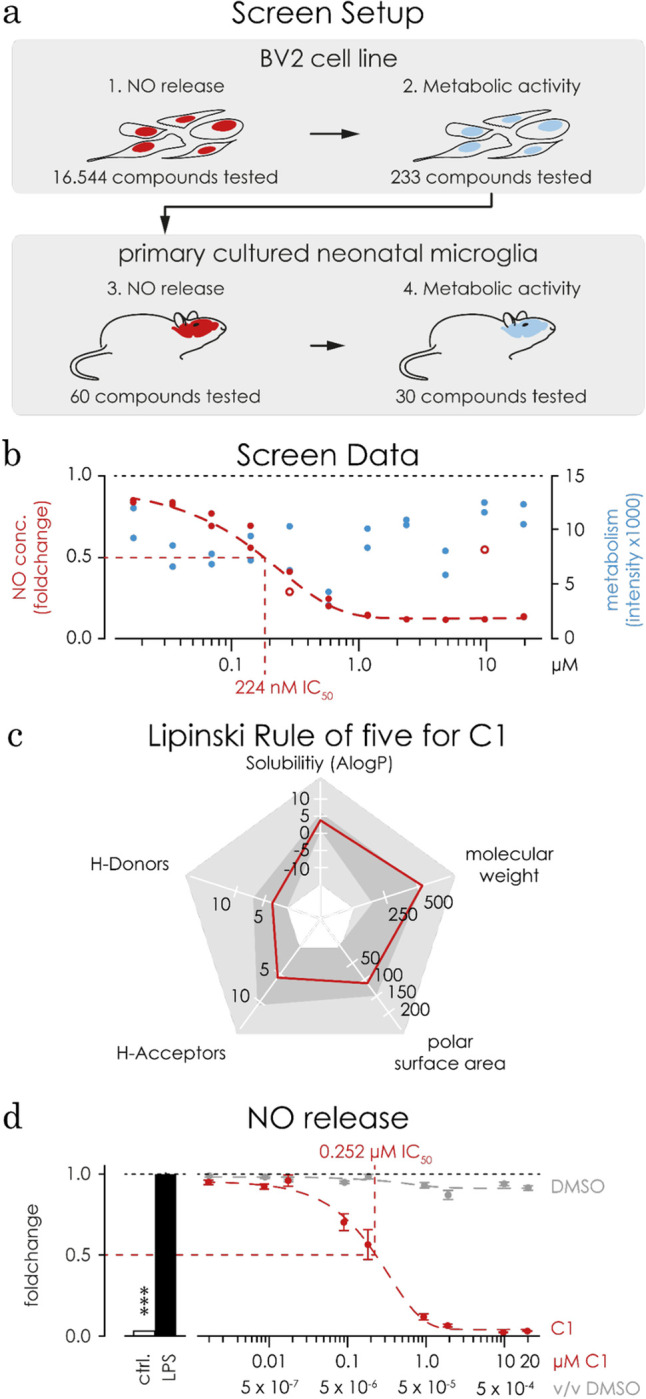
C1 reduces LPS induced microglial NO release in a dose dependent manner. **a)** Representative illustration of the HTS setup. Initially (1.), 16 544 compounds were tested for their ability to reduce LPS-induced NO-release in BV-2 cells. (2.) The best 233 compounds were tested for their impact on metabolic activity. (3.) 60 compounds passed and were further analysed in primary microglial cells. (4.) 30 out of 60 reduced LPS induced NO release, and showed no impact on metabolic activity. Out of the 30 compounds C1 was chosen and further analysed. **b)** The data obtained from the screen showed a dose-dependent reduction of the concentration of NO in the medium (shown in red). The calculated IC_50_ value is 224 nM (vertical red dotted line). Two data points of the NO assay, marked as red open circles, were automatically excluded using a robust fit outlier model in GraphPad prism. The metabolic activity remained stable (shown in blue). **c)** C1 fulfils 4 of the 5 “Lipinski rule of five”, that include the number hydrogen bond donors (Lipinski ≤ 5, C1 = 2), and the hydrogen bond acceptors (Lipinski ≤ 10, C1 = 6), polar surface area (Lipinski 140 ≤ Å2, C1 = 106.2 Å2), solubility (Lipinski ≤ 5 AlogP, C1 = 4.7 AlogP), but is slightly beyond the limit for the molecular weight (Lipinski ≤ 500 g/mol, C1 = 517.6 g/mol). **d)** Transferred to a laboratory scaled setup, primary cultured microglia were treated with C1 or DMSO for 1 hour followed by an additional stimulation with 1 μg/mL LPS for 48 hours where we confirmed C1 ability to reduce LPS induced NO release dose-dependently (concentrations ranged from 0.0002 μM to 20 μM, in red). DMSO alone did not affect the NO release (concentrations ranged from 0.6x10^-6^ v/v up to 1x10^-3^v/v, in grey). The calculated IC_50_ value was 252 nM thus only slightly higher than in the BV-2 cell line.

First, a high throughput screen (HTS, **Fig 1A**) of a small molecule library containing 16 544 compounds was performed in BV2 microglia cells to identify compounds that inhibit the lipopolysaccharide (LPS)-induced NO release in microglia. The NO concentration in the supernatant was measured using a modified Griess assay [46]. The assay plates showed a signal separation suitable for HTS with a mean Z’-factor of 0.75 (based on the signals of LPS-induced versus plain medium). The compounds were pre-selected to cover a broad diversity of chemical structures [32] and the “Lipinski rule of 5” was taken into account to ensure general bio availability [47, 48]. BV-2 cells were pre-treated with 5 μM of each compound for 1 hour and 1 μg/mL LPS was added for an additional 48 hours. Out of 16 544 compounds, 503 presented a Z-score < -5. Second, the 352 most effective compounds were picked for concentration-dependent validation (19.5 nM up to 20 μM) and 233 compounds were identified. Third, we tested the effect of each compound on BV-2 cell viability, using the AlamarBlue assay. 60 out of 233 compounds reduced the NO release without compromising cell viability. Fourth, 30 out of 60 compounds reduced the LPS-stimulated NO concentration in primary neonatal microglia. Fifth, we reduced the list to 4 candidates, which reduce LPS-induced NO-release in a dose-dependent manner, while not interfering with the cell viability of primary microglia. Finally, out of those 4 compounds, C1 presented with the best IC_50_ value (224 nM) in reducing NO release (indicated by the vertical red dashed line, **Fig 1B**). Additionally, C1 shows a general calculated drug-ability, fulfilling most of the “Lipinski rule of 5” (**Fig 1C**).

In a low scale laboratory setting (using a 96 well plate, **Fig 1D**), microglia were treated for 1 hour with C1 (2 nM up to 20 μM) and subsequently stimulated with 1 μg/mL LPS for 48 hours. The IC_50_ value was slightly higher in primary microglia than in BV-2 cells (224 nM in the HTS versus 252 nM; **Fig 1D**, red dotted line), and C1 did not affect cell viability (**S1A Fig**, red dotted line). We observed a 31% increase in metabolic activity in LPS-stimulated microglia (in black) compared to the unstimulated control (in white, p < 0.0001) but never to levels below the negative control (**S1A Fig**). DMSO was used as the solvent for the compound and did not affect the NO release (**Fig 1D**, grey dotted line) or the cell viability of microglia (**S1A Fig**, grey dotted line). Additionally, C1 and DMSO do not quench the NO concentration after incubating NO enriched supernatant taken from LPS-stimulated microglia (**S1B Fig**).

Finally, NO release and metabolic activity of naïve microglia are not affected by C1, given that 3 different concentrations of C1 (0.025 μM, 0.25 μM, or 2.5 μM, in red for NO release, in blue for metabolic activity) or their corresponding concentrations of DMSO (1.25x10^-5^, 12.5x10^-5^, or 125x10^-5^ v/v, all in grey) for 48 hours did neither change the NO release (**S2A Fig**) nor interfered with metabolic activity (**S2B Fig**) of microglia.

### C1 reduces nitric oxide release induced by IFNγ or PolyIC

Alternatively, to mimic a bacterial infection using LPS, microglia can be challenged *in vitro* using IFNγ to mimic a Th1 cell response [49] or PolyIC to mimic an anti-viral response [50]. To determine the compound’s effect on already stimulated microglia (LPS, IFNγ, and PolyIC) we normalized the results of each individual experiment to its stimulated control.

**Fig 2:**
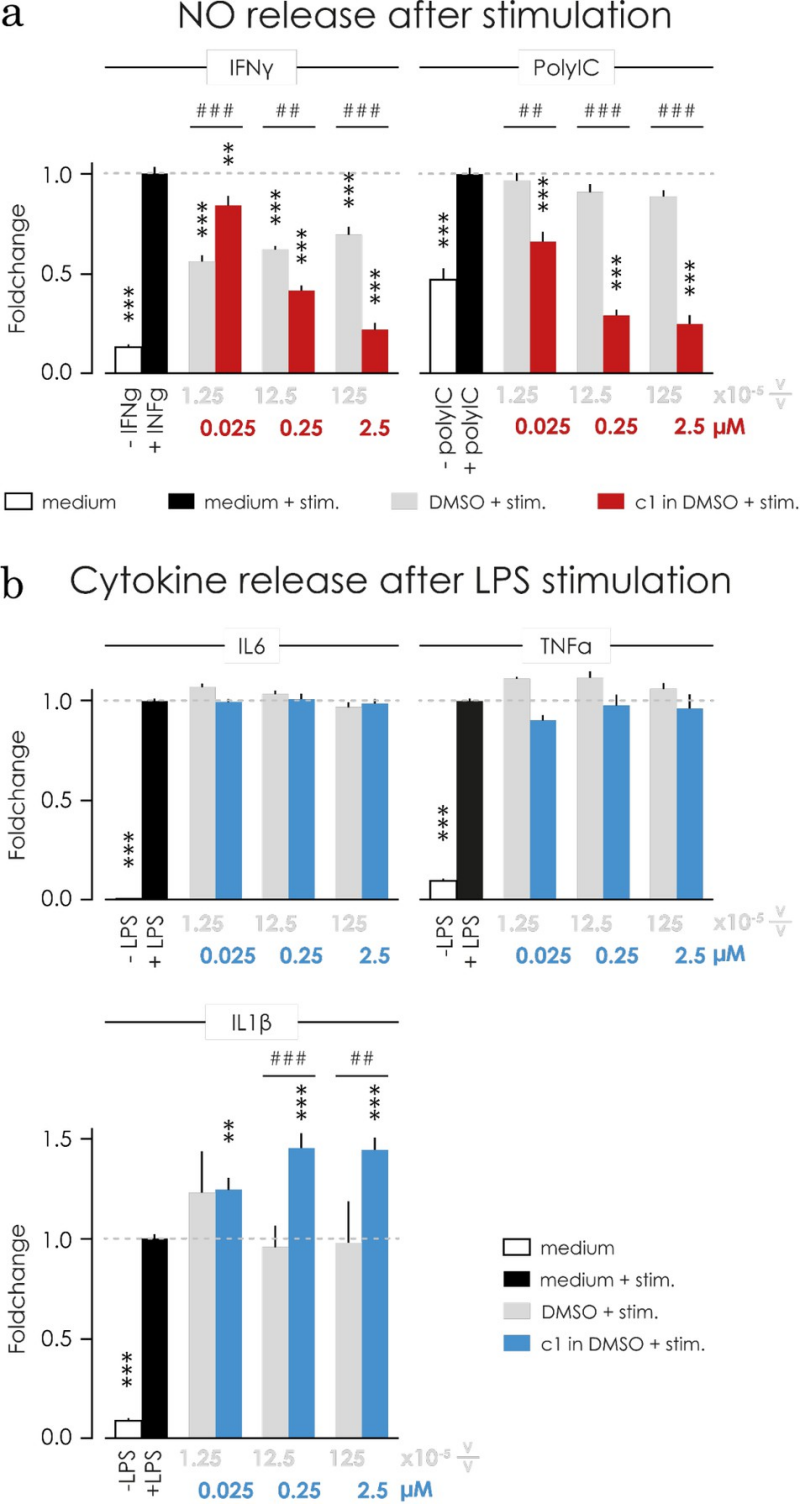
In microglia, C1 reduced NO release triggered by IFNγ and PolyIC, and did not affect LPS induced cytokine release. **a)** Stimulation of microglia with IFNγ (100 ng/mL) for 48 hours (left, in black) and PolyIC (100 μg/mL) for 24 hours (right, in black) caused a significant increase in the production of NO (p<0.0001) in comparison with unstimulated control (white bar). Treatment with C1 caused a dose-dependent decrease of NO release in primary cultured microglia upon stimulation with IFNγ (left, in red) and PolyIC (right, in red). Incubation with DMSO (in grey) had no effect on the NO release under PolyIC stimulated condition, however, DMSO decreased the NO level in IFNγ-stimulated microglia (p<0.0001) but not dose dependently. **b)** Pre-treating microglia for 1 hour with C1 followed by an 48 hour stimulation with LPS (1μg/mL, black bar) increased the concentration of the pro-inflammatory cytokines IL6, TNFα and IL1β compared to the negative control (plain medium in white). Treatment with C1 (in blue) did not have a significant effect for the LPS-induced IL6 and TNFα release, but increased the release of IL1β at all tested concentrations (0.025 μM: p = 0.0037, 0.25 μM: p < 0.0001, 2.5 μM: p < 0.0001). The respective DMSO controls (grey bar) did not significantly affect the IL6, TNFα and IL1β release. *** p<0.001 ** p<0.01 comparing to stimulated control; ### p<0.001 ## p<0.01 compared to DMSO (1way ANOVA followed by Bonferroni’s post-hoc test).

Stimulating primary microglia with IFNγ (100 ng/mL, **Fig 2A**, left panel) for 48 hours evoked a significant increase in NO concentration (p<0.0001) which was blocked in a dose dependent manner by 1h of pre-treatment with C1. The solvent DMSO alone also decreases the IFNγ-induced NO concentration by approximately 40% (**Fig 2A**, grey bars), in a non-dose-dependent fashion and never to the same extent as C1.

When stimulating microglia with PolyIC for 48 hours we observed a decrease in the cell viability (measured with the AlamarBlue assay, data not shown). We thus proceeded to stimulate primary microglia for 24 hours with PolyIC (100 μg/mL, **Fig 2A**, right panel) and observed an increase in the NO concentration in the supernatant (-PolyIC, p<0.0001). Pre-treatment with C1 resulted in the ablation of NO production in a dose dependent manner to values below the plain medium control (-PolyIC). DMSO has no effect on the PolyIC induced NO levels. Although we do not investigate the stand-alone effect of DMSO on microglia’s NO production, it is worth mentioning that it is impacted in IFNγ (**Fig 2A)** but not in LPS (**S2A Fig**) and PolyIC (**Fig 2A**) stimulated microglia. For this reason, we always show both the plain medium and the DMSO control. We consider the most meaningful comparison to be the one with the stimulated control (+LPS, +IFNγ, or +PolyIC) since we are investigating the inhibitory potential of C1.

### C1 does not interfere with the LPS-induced IL6 and TNFα release

Upon pro-inflammatory stimulation, microglia produce pro-inflammatory cytokines, such as IL1β, IL6, and TNFα [7, 8, 51]. Pre-treatment of microglia with C1 for 1h followed by stimulation with 1 μg/mL LPS for 48h, did not induce changes in the concentration IL6 and TNFα (**Fig 2B**) and induced increased production of IL1β (25 nM: p = 0.0037; 250 nM and 2.5 μM: p<0.0001, **Fig 2B**). Moreover, C1 alone does not alter the levels of IL1β, IL6, and TNFα (**S2C Fig**) indicating that the observed increase in the IL1β concentration might be the result of a synergistic effect of the stimulation with LPS and C1. DMSO does not affect cytokine release, neither in stimulated (**Fig 2B**, shown in grey) nor unstimulated microglia (**S2A–S2C Fig**, shown in grey).

### C1 acts as a chemoattractant, but did not affect phagocytosis activity

**Fig 3:**
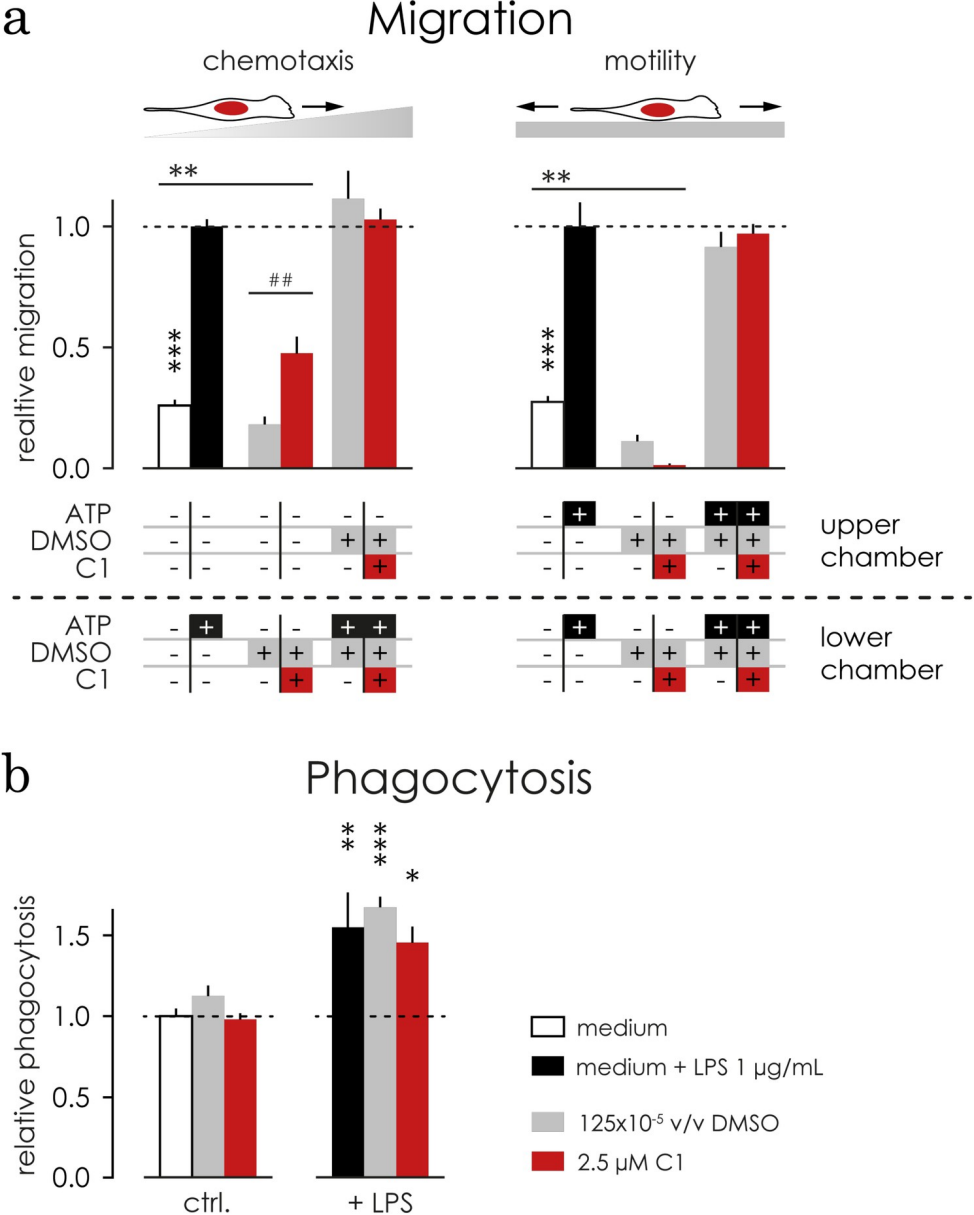
C1 acts as a chemoattractant but did not influence microglial motility and phagocytosis. **a)** C1 affected microglial chemotaxis. The results were normalized to ATP induced (black bars) chemotaxis or motility, respectively. A gradient of 2.5 μM C1 (in red) increased chemotaxis significantly compared to plain medium (in white, p = 0.0036) and DMSO (in grey, p = 0.0012). 2.5 μM C1 did not affect ATP induced chemotaxis in microglia. **b)** C1 (in red) significantly decreased microglial motility when compared to plain medium (white, p = 0.0018) but not to DMSO (grey). ATP induced motility did not change in the presence of C1 or DMSO. *** p<0.001 ** p<0.01 comparing to stimulated control, ### p<0.001 ## p<0.01 comparing to DMSO (1way ANOVA followed by Bonferroni’s post-hoc test). **c)** The phagocytic activity both under basal conditions (left, ctrl.) and LPS stimulated conditions (right, +LPS) was assessed using a FACS-based protocol. Microglia were pre-treated with 2.5 μM C1 in DMSO (red) or its corresponding concentration of DMSO (in grey,125x10^-5^ v/v) and compared to the control medium. Neither C1 nor DMSO altered microglial phagocytosis significantly compared to plain medium (white). Upon stimulation with 1 μg/mL LPS for 24 hours phagocytosis increased significantly around 60% compared to their own unstimulated control (plain medium: +55% p = 0.0066, DMSO: +67% p = 0.0068, C1: +45% p = 0.0163, black striped bars). Within LPS stimulated conditions there were no significant differences. *** p<0.001 ** p<0.01 compared to plain medium control (non-matching 2way ANOVA followed by Sidak test).

Pro-inflammatory stimulation of microglial cells often results in increased motility, chemotaxis and phagocytic activity [6–8]. Leveraging the Boyden-chamber assay in the presence of a stimulus gradient, we can assess microglia chemotaxis. In addition, when there is no gradient and the substance of interest is equally distributed through the chamber, we can determine the effect on cell motility (**Fig 3A**). To assess the chemoattractant potential of C1, we applied a gradient with 2.5 μM C1 and used the corresponding 125x10^-5^ v/v DMSO and plain medium as controls. The number of cells migrating towards C1 was significantly higher compared to plain medium or to the DMSO control (p = 0.0079, **Fig 3A**), indicating that C1 has chemoattractant properties. The ATP induced chemotaxis (set to 100%) was not changed by C1 (103%) or DMSO (112%) (**Fig 3A**).

Motility was assessed in the presence of 2.5 μM C1, DMSO (125x10^-5^ v/v), or plain medium on both sides of the Boyden-chamber. C1 increased microglia motility when compared to the plain medium (p = 0.0018) but not DMSO control and did not affect the ATP induced motility in microglia (**Fig 3B**). We hereby conclude that C1 acts as a chemoattractant in microglia.

We also determined the phagocytic activity both under basal conditions and after LPS stimulation in the above referred conditions and even though LPS induces a 30% increase in microglia phagocytic activity (p = 0.0058, **Fig 3C**). C1 and DMSO did not affect phagocytosis activity.

### C1 decreases NO release in microglia 24 hours after LPS stimulation

**Fig 4:**
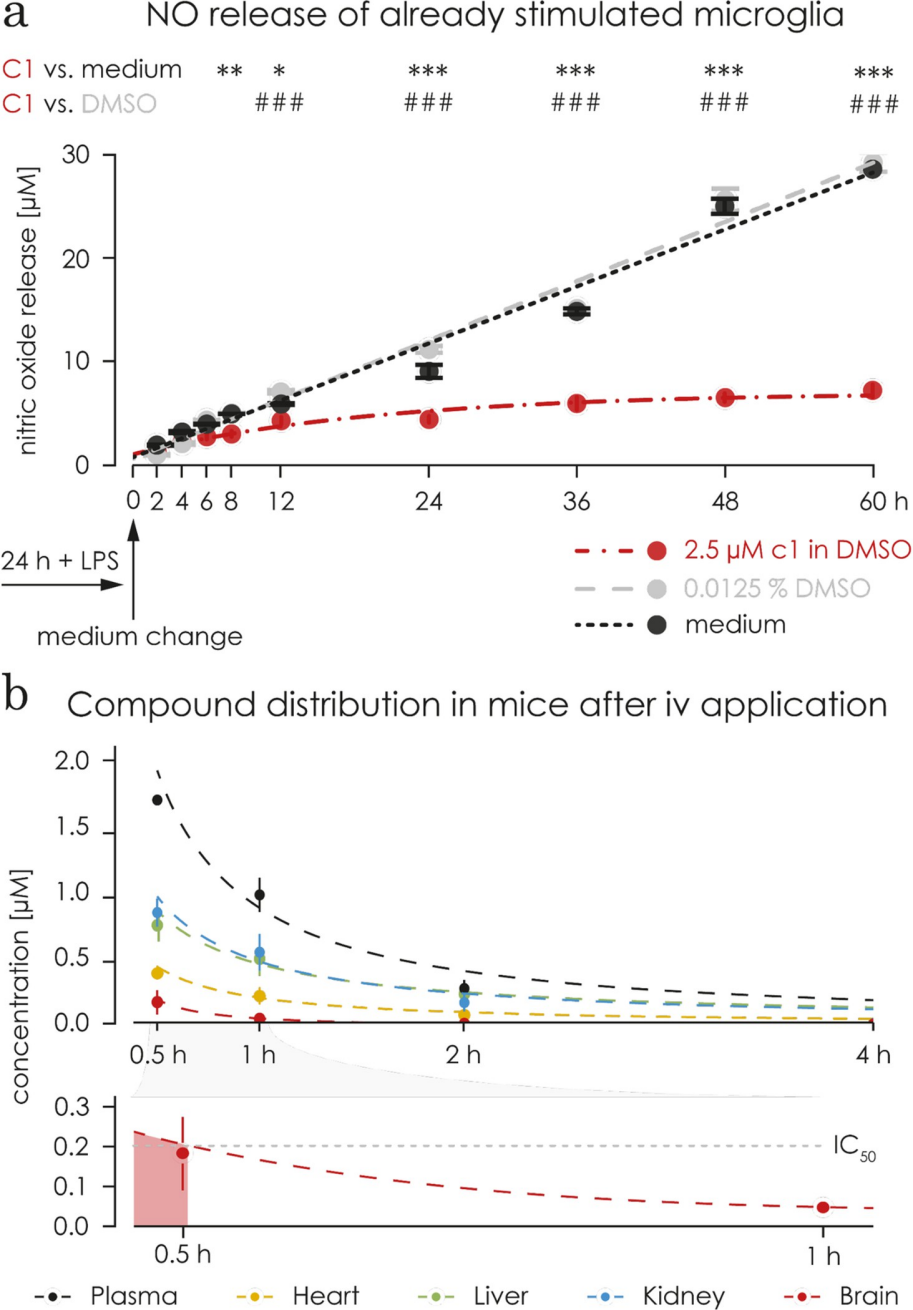
C1 showed an effect on pre-stimulated microglia and passed the blood brain barrier. **a)** Microglia were stimulated for 24 hours with 1 μg/mL LPS before being treated with 2.5 μM C1 (in red), the corresponding concentration of DMSO (125x10^-5^ v/v) in plain medium (in grey) or with plain medium only (in black). NO concentration increased linearly in the supernatant of untreated microglia, reaching 28 μM (± 1.036 SEM) after 60 hours. DMSO treatment did not change this linear increase, reaching 29 ± 1.119 μM at the same time point. Treatment of microglia previously stimulated with LPS with C1 led to an increase in NO concentration to a lower extent over time compared to DMSO and plain medium treatment. The increase in NO concentration followed a one-phase association (R^2^ of 0.6498) and reached a calculated plateau of 7 μM (95% confidence interval 5.999 to 8.127). After 8 hours of treatment with C1, the level of NO was significantly reduced compared to plain medium (p = 0.006), and after 12 hours compared to the cells treated with DMSO (p < 0.0001). *** p<0.001 ** p<0.01 comparing to plain medium control, ### p<0.001 ## p<0.01 comparing to DMSO control (non-matching 2way ANOVA followed by Sidak test). **b)** The upper graph shows the distribution of C1 in different organs after a single intravenous injection into healthy male mice (5 mg/kg, n = 3). The concentration of C1 in blood plasma (in black), heart (in red), liver (in green), kidney (in blue), and brain (in yellow) was monitored for 4 hours (samples were collected after 30 min, 1 h, 2 h, and 4 h). The lower graph shows the C1 level in the brain amplified from the upper graph. It reached a concentration above the IC_50_ value during the first 0.5h.

To mimic a situation closer to a therapeutic use, we investigated the effect of C1 on already stimulated microglia. Microglia were stimulated with LPS for 24 hours, the media was changed and 2.5 μM C1, 125x10^-5^ v/v DMSO or plain medium were added to determine the temporal evolution of NO production. The NO concentration in the supernatant of cells in plain medium or DMSO increased in a linear fashion, reaching 28 μM (±1.036) after 60h (**Fig 4A**). Treatment with C1 reduced the NO production, after 8 hours of treatment when compared to plain medium (p = 0.006), and after 12 hours when compared to the DMSO (p<0.0001). The increase of the NO concentration after C1 treatment followed a one-phase association (R^2^ of 0.6498) and reached a calculated plateau of 7 μM (**Fig 4A**) compared to 28 μM for DMSO or plain medium. Therefore, C1 is effective in decreasing NO production of microglia 24 hours after LPS stimulation.

### C1 has similar effects on macrophages

Microglia and macrophages share many of their immune properties. Therefore, we evaluated the impact of C1 on the release of NO, IL1β, IL6, and TNFα on bone marrow derived macrophages [52, 53] isolated from adult mice and pre-treated for 1 hour with C1 (0.025 μM, 0.25 μM, or 2.5 μM), its corresponding concentration of DMSO (1.25x10^-5^, 12.5x10^-5^, or 125x10^-5^ v/v), or plain medium before a stimulus was added. The NO release was measured after stimulation with either 1 μg/mL LPS for 48 hours, 100 μg/mL PolyIC for 24 hours or 100 ng/mL IFNγ for 48 hours. C1 reduced the NO release in a dose dependent manner for all three stimuli (**S3 Fig)**. 2.5 μM C1 decreased NO concentration to the level of plain medium (p>0.9999), IFNγ (p = 0.1253) and PolyIC (p = 0.9751). DMSO did not alter NO release in bone marrow derived macrophages stimulated with LPS or PolyIC (**S3A and S3B Fig**). However, it reduced the IFNγ-induced NO release in these cells independently of the applied dose (**S3C Fig**), similarly to IFNγ-stimulated microglia (**Fig 2A**).

All tested concentrations of C1 significantly decreased the metabolic activity of LPS-stimulated macrophages compared to plain medium control, but only 2.5 μM reached significance compared to its own DMSO control (125x10^-5^ v/v; p = 0.0310, **S3D Fig**). The metabolic activity of PolyIC (100 μg/mL) stimulated macrophages was decreased significantly by the lowest compound concentration (0.025 μM) compared to plain medium (p = 0.138) and to its DMSO control (1.25x10^-5^ v/v, p = 0.0053) (**S3E Fig**). 0.25 μM and 2.5 μM C1 decreased the metabolic activity of IFNγ-stimulated macrophages compared to plain medium, but not to its DMSO control (**S3F Fig**).

The treatment neither with C1 nor with DMSO showed any significant changes in the release profile of IL1β and IL6 48 hours after LPS stimulation **(S3G Fig)**. There is, however, a dose independent decrease in the concentration of TNFα after treatment with C1 and with DMSO. Macrophages respond similarly like microglia to C1 treatment, albeit with a slightly different metabolic profile. In order to test the effect of the compound on other cell types of the brain, we next used astrocytes and oligodendrocytes.

### Metabolism, proliferation and cell death of astrocytes and oligodendrocytes are not affected

We evaluated the metabolic activity, proliferation and cell death of primary cultured neonatal microglia, primary cultured neonatal astrocytes and the oligodendrocyte cell line OLN-93 under physiological conditions. All cells were treated with 2.5 μM C1 in DMSO or the corresponding DMSO concentration (125x10^-5^ v/v) for 48 hours without any additional stimulation followed by either an AlamarBlue assay to measure the metabolic activity, or a propidium iodide based proliferation and cell death assay. Cell death information is given in percentage of the total amount of cells. Under physiological conditions, C1 and DMSO induced a 15% increase of the metabolic activity of microglia (C1: p = 0.0402, DMSO: p = 0.0439, **S4A Fig**). However, there were no significant differences between treatment with C1 and DMSO. Astrocytic metabolic activity was not affected by C1 or DMSO (**S4A Fig**). However, C1 induced a significant increase of OLN-93 cells compared to DMSO (p = 0.0431, **S4A Fig**). C1 induces a significant increase of microglia proliferation to 117% compared to plain medium (p = 0.0154) and DMSO (p<0.0001). DMSO, but not C1, increased the proliferation of astrocytes compared to plain medium (p = 0.0060). Neither C1 nor DMSO changed the proliferation of OLN-93 cells (**S4B Fig**). The percentage of dead microglia increased significantly in the presence of DMSO compared to plain medium (p = 0.0096). Treatment with C1 in DMSO brought this value back to the level of the plain medium. The difference between the elevated DMSO death rate and C1 was significant (p = 0.0201). C1 and DMSO showed no significant effect on astrocytes and OLN-93 cell death rate (**S4C Fig**). The effects of the compound on the metabolism, proliferation and death of brain-derived cells are minimal and thus we felt safe to conduct an *in vivo* experiment.

### C1 passes the blood brain barrier in an *in vivo* mouse model

The bioavailability was evaluated *in vivo* in CD1 male mice performed by Touchstone Biosciences. C1’s tissue distribution was monitored in the blood plasma, kidney, liver, heart and brain, over a period of 4 hours (30 min, 1, 2, and 4 hours) after an intravenous injection of 5 mg/kg into a cohort of 3 mice (**Fig 4B**). After 30 minutes, the concentration in the brain was 0.1 μM/g tissue. The concentration continuously decreased and reached a concentration of 2.4 nM/g tissue, 4 hours after the injection. Within the 24-hour analysis period no health issues were reported and all mice survived.

### C1 improves extrapyramidal motor skills and laterality in an *in vivo* model of ischemic injury

**Fig 5:**
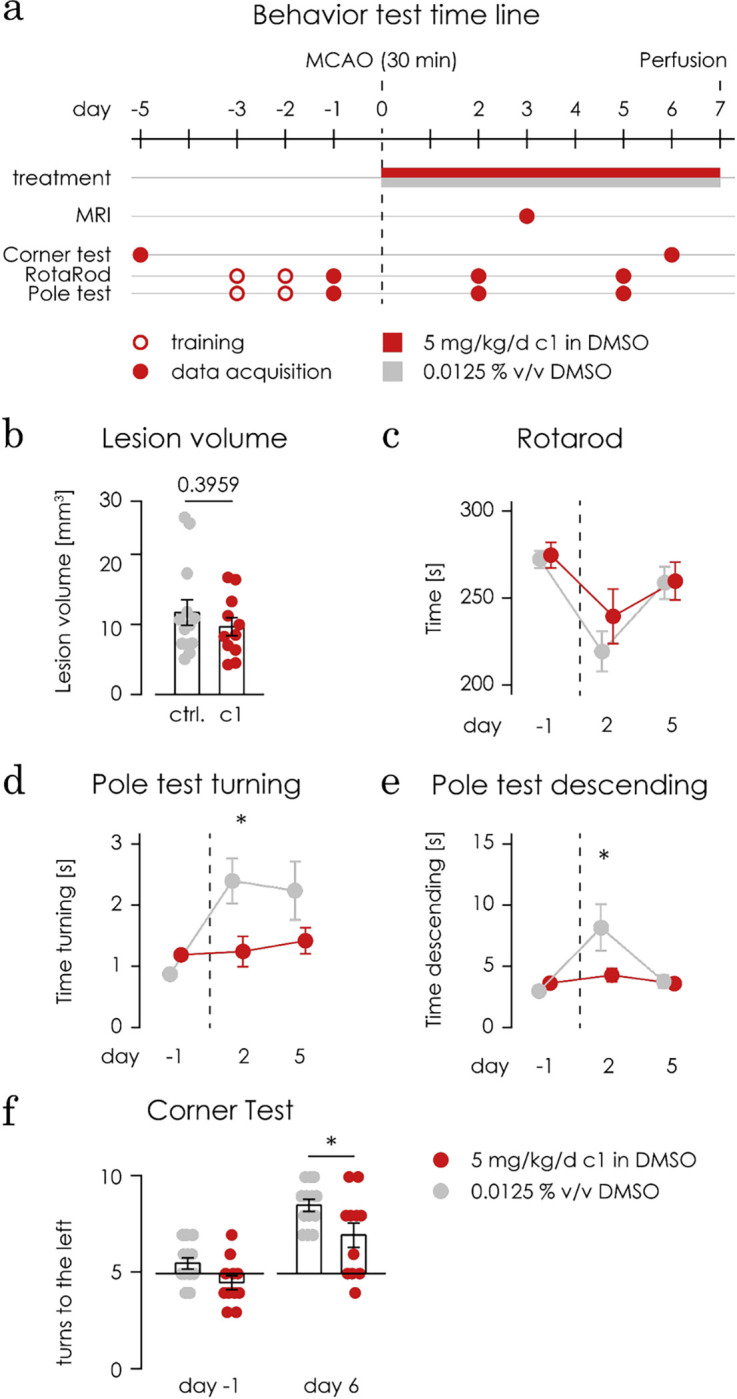
C1 improves behavioural deficits after mild brain ischemia. **a)** The scheme shows the experimental paradigm. Male C57Bl/6 mice were submitted to 30 minutes of middle cerebral artery occlusion (MCAo), as a model for mild ischemic injury (indicated by the dashed vertical line). From the day of the MCAo surgery until day 6 after MCAo, mice were injected i.p. daily with 5 mg/kg of C1 diluted in 125x10^-5^ v/v DMSO as a vehicle (n = 11) or with just the vehicle (125x10^-5^ v/v DMSO in PBS) as control (n = 13). The motor coordination (accelerated Rotarod) and extrapyramidal motor locomotion (pole test) were assessed at day 2 and 5 after MCAo, mice were trained for the both tests on day 3 and 2 before MCAo and baselines were taken on the day before MCAo. The corner test was used to test mice’s laterality on day 6 after MCAo by counting the amount of turns to the left in a total of 10 trials which was then compared to each mouse individual baselines, taken 5 days before MCAo. On day 3 after MCAo, MRI scans were performed to determine the volume of the ischemic lesion. **b)** The treatment of mice with C1 (in red) did not cause a significant difference (p = 0.3959) in the volume of the ischemic lesion, measured with MRT in comparison with the controls (in grey), using an unpaired t test. **c)** There are no differences between the treated group (in red) and the controls (in grey) in the Rotarod test, using a 2way ANOVA test. **d)** During the pole test, the C1-treated group was significantly (in red, p = 0.0253) faster than the control group in turning 180° upside down (1.175s versus 2.328s) on day 2 after MCAo. No differences were observed on day 5 after MCAo. A 2way ANOVA test was used to test for statistical relevance. * p < 0.05. **e)** Additionally during the pole test, the treated group was significantly faster at descending the pole (p = 0.0145; treated in red: 6.592s versus controls in grey:10.47s) on day 2 after MCAo. No differences were observed on day 5 after MCAo. A 2way ANOVA test was used to test for statistical relevance. * p < 0.05. **f)** The animals treated with C1 (in red, in the right) performed fewer turns to the left (mean 7.00 in 10 turns, p = 0.0213) in comparison to controls (in grey, 8.54 in 10 turns to the left), using a 2Way ANOVA statistical test * p < 0.05.

Since C1 was potent to dampen NO release *in vitro* without toxic side effects *in vitro* or *in vivo*, and crossed the blood brain barrier, we felt confident to test its potential as a treatment in an animal model for ischemia. We used 30 minutes of middle cerebral artery occlusion (MCAo) in male C57Bl/6 mice [54]. Mice were injected i.p. daily with 5 mg/kg of C1 diluted in 125x10^-5^ v/v DMSO in PBS (n = 11) or with just DMSO in PBS (n = 13) as control for the 7 days after MCAo. The experimental time course of the behavior tests, MCAo and the treatment is illustrated in **Fig 5A**.

On day 3 after MCAo, we assessed the lesion volume using MRT. No significant difference in lesion volume was found between the groups (**Fig 5B**). As a functional readout for neurological parameters, the motor coordination was tested with the accelerated Rotarod test, and their extrapyramidal motor locomotion was assessed using the pole test. Before MCAo, the mice of both groups were trained both in the Rotarod and in the pole test for 2 days and baselines were taken on the third day. On day 2 and 5 after MCAo, motor deficits were assessed, and the results were compared to the baseline (**Fig 5C–5F**). No significant difference was found between the groups in the Rotarod test (**Fig 5C**). However, in the pole test, the treated group was significantly (p = 0.0253) faster than the control group in performing the turn (1.175s versus 2.328s, **Fig 5D**) and in descending the pole (p = 0.0145; 6.592s versus 10.47s, **Fig 5E**) on day 2 after MCAo, showing improved extrapyramidal motor locomotion.

Another important neurological parameter is laterality (the pathologic preference to turn to one side). Mice’s laterality was tested with the corner test on day 6 after MCAo and the amount of turns to the left in a total of 10 trials was compared for each mouse baseline, taken 5 days before MCAo. The animals treated with C1 display less lateral preference (7.00 in 10 turns to the left, p = 0.0213) in comparison to controls (8.538 in 10 turns to the left, **Fig 5F**).

### C1 exists in 2 stereoisomers, C1-*S* and C1-*R*, with different impacts on the LPS-induced NO production

**Fig 6:**
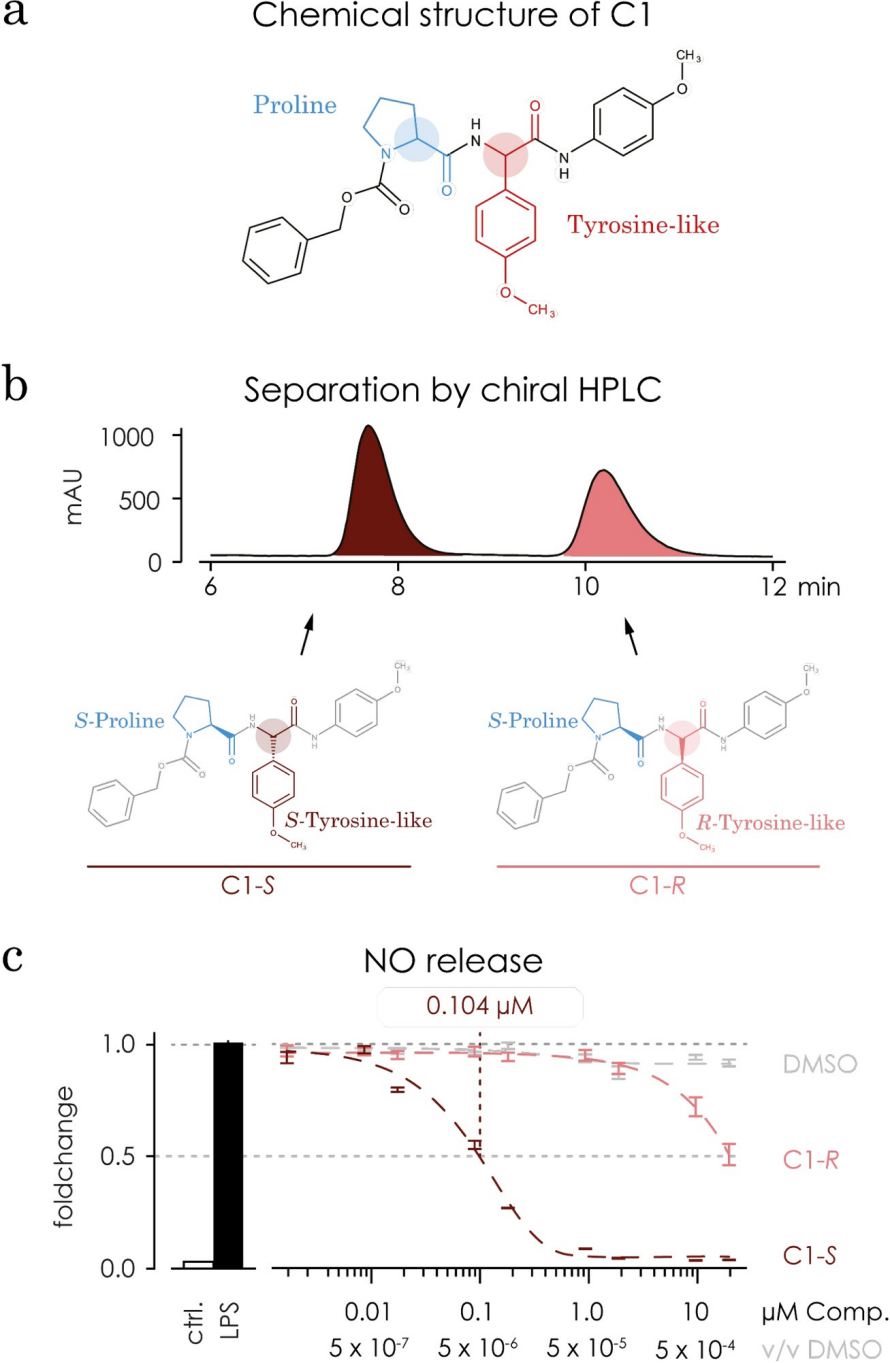
C1 exists in 2 stereoisomers. **a)** C1’s structure inherits 2 stereo centres, both located in the backbone of the peptide bonds, one in the amino acid proline (shown in blue) and the other one in the amino acid phenylglycine (shown in red). The stereo centre in proline amino acid is defined in its *S* configuration. The stereo centre in the amino acid phenylglyicne is not defined and consists of both *S* and *R* configurations. Both configurations are shown below on b. **b)** The stereoisomeric mixture of C1 was separated into its stereoisomers using a chiral HPLC, resulting in two distinct peaks. The area under the curve for both peaks are 43% (shown in dark red, C1-*S*) and 57% (shown in light red, C1-*R*) in relation to the total area. **c)** Repeating the assays shown in Fig 2 the stereoisomers C1-*S* (in dark red) and C1-*R* (in light red) showed different effects on the NO release compared to C1, but not in the metabolic activity or NO scavenging. Microglia were treated with C1-*S*, C1-*R* or DMSO for 1 hour followed by an additional stimulation with 1 μg/mL LPS for 48 hours. The concentration of the compounds ranged from 0.0002 μM to 20 μM and for DMOS (in grey) from 0.6x10^-6^ v/v up to 1x10^-3^v/v. The NO concentration was tested using a Griess assay. The calculated IC_50_ value for C1-*S* was 104 nM (95% CI = 0.09491 to 0.1152) and for C1-*R* 43.29 μM (95% CI = 8.841 to +infinity).

C1 is a small peptide-like molecule (**Fig 6A**). Its structure inherits 2 stereo centres located in the backbone of the peptide bonds, one in the amino acid proline (defined in its *S* configuration) and the other one in the amino acid phenylglycine (not defined, consisting of both *S* and *R* configurations). Both diastereomers were separated using a chiral HPLC, resulting in two distinct peaks, named C1-*S* (43% of total area) and C1-*R* (57% of the total area, **Fig 6B**). Re-synthesis of both diastereomers of C1 was performed in 5 steps starting with either the (S) or the (R) enantiomer of 4-methoxyphenylgycine and using standard peptide synthesis and protecting groups. Optimized control of coupling reagents secured the integrity of the desired stereo centres analysed with chiral HPLC, H-NMR and C-NMR. The re-synthesised enantiomers could be allocated to the two peaks found in the HPLS of the stereoisomeric mixture. The data for the synthesis are found in the section 1 of S1 File.

We evaluated the reducing effect on LPS-induced NO-release in microglia for both diastereomers individually. Microglia were treated for 1 hour with C1-*S* or C1-*R* (1 nM up to 20 μM) and subsequently stimulated with 1 μg/mL LPS for 48 hours. Afterwards NO concentration was measured using the Griess assay. The IC_50_ value of C1-*S* was at 104 nM (41% of the IC_50_ value of the diastereomeric mixture C1) and the IC50 of C1-*R* was 43.29 μM, which is outside the tested concentration range of the diastereomeric mixture C1 (**Fig 6C).** Treatment with C1-*S* (1 nM to 20 μM) showed significant decrease of NO compared to plain medium while 10 nM and 10 μM showed a non-significant decrease. The resulting metabolic activity ranged between 83% (20 μM) and 101% (10 nM). C1-*R* decreased the metabolic activity significantly only at concentrations of 1 μM (p = 0.0188) and 20 μM (p = 0.0023). The measured values ranged from 95% (20 μM) to 102% (2 μM). Furthermore, neither of the two diastereomers quenched NO concentration. Compared to plain medium, the measured NO concentrations (2 to 1 μM) ranged from 98% to 105% for C1-*S* and 98% to 102% for C1-*R*.

### C1-S acts on the posttranscriptional level

**Fig 7:**
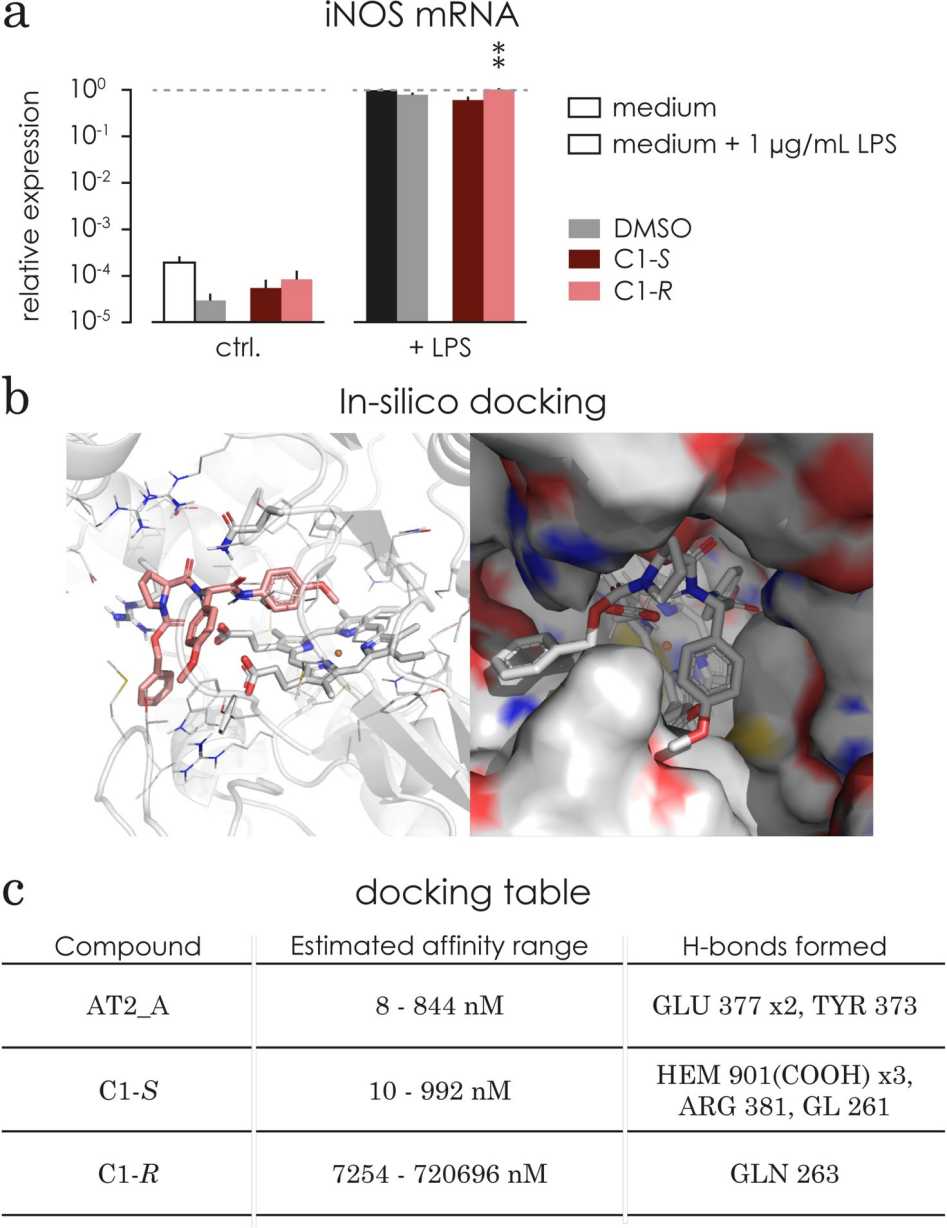
C1 does not change *iNOS* mRNA levels and C1-*S* has a higher affinity than C1-*R* in an *in-silico* docking calculation. **a)** The relative expression of *iNOS* mRNA was evaluated under basal conditions and LPS stimulated (1 hour compound treatment + additional 24 hours 1 μg/mL LPS) conditions. Under basal conditions *iNOS* mRNA level was barely detected. DMSO, C1, C1-*S* and C1-*R* had no significant effect on the basal expression of *iNOS* mRNA. Upon 1 μg/mL LPS stimulation *iNOS* mRNA expression increased more than 5000 fold independently of the treatment (induced vs basal: p < 0.0001). Within the stimulated conditions, treatment with DMSO, C1, C1-*S* and C1-*R* did not significantly alter *iNOS* expression. All treatments led to a significant increase in iNOS mRNA upon LPS stimulation C1: p<0.0001, C1-*S*: p = 0.0236, C1-*R*: p<0.0001, DMSO: p<0.0001). *** p<0.001 comparing to plain medium control (non-matching 2way ANOVA followed by Sidak test). **b)** Predicted binding mode of compounds C1-*S* in the binding pocket of iNOS structure (PDB 3e7g). Protein binding site shown in cartoon representation (colour coding by element oxygen = red, nitrogen = blue, sulphur = yellow, carbon = grey). Heme as well as two hydrogen bond forming residues shown as sticks. Compound C1-*S* represented as sticks in salmon. Right: Surface representation shown from a different angle. Figure generated using PyMol [40]. **c)** Estimated affinity ranges (using SeeSAR) for the co-crystallized ligand (AT2_A, re-docking) as well as compounds C1-*S* and C1-*R* sorted by ascending mean estimated affinity (the lower the value the better) docked into the binding site of iNOS (PDB structure 3e7g^39^).

It is known that *iNOS* is regulated on the transcriptional level [11]. Under physiological conditions *NOS2* mRNA is barely detectable, while upon a pro-inflammatory stimulus the transcription of *NOS2* mRNA is upregulated. To determine C1 effect on *NOS2* expression, microglia were pre-incubated with C1-*S* or C1-*R* diluted in DMSO (2.5 μM), DMSO alone (125x10^-5^ v/v) or plain medium for 1 hour, followed by additional 24 hour incubation with or without 1 μg/mL LPS. No differences were observed after treatment with C1-*S*, C1-*R* or DMSO alone. However, stimulation with LPS increased the *NOS2* expression by 5000-fold (p<0.0001, **Fig 7A**), in comparison to plain medium and even though treatment with C1-*S* or DMSO has the same effect as plain medium, treatment with C1-*R* increased *NOS2* expression significantly above the LPS stimulated plain medium control (p = 0.0083).

### Based on *in-silico* protein-ligand docking calculations, C1-*S* shows a higher predicted affinity towards iNOS compared to C1-*R*

In microglia, the LPS induced NO release is regulated by iNOS. In order to obtain information about potential binding mechanisms of C1-*S* to iNOS, structure-based docking calculations were carried out. Docking was used as an efficient computational predictor of a possible binding mode and an estimated affinity using a scoring function [55, 56]. Redocking of the co-crystallized ligand (ID: AT2_A) to the WT human iNOS structure (PDB: 3e7g) (35) showed estimated affinity values comparable to the experimentally determined IC_50_ value of 350 nM, and root mean square deviation (RMSD) between the co-crystallized and redocked ligand AT2_A of 0.344 Å (calculated with DockRMSD) [57]. Both indicate that the protein structure can be used for further docking and scoring experiments with this method (**Fig 7B**).

A binding pose of compound C1-*S* was generated with an estimated affinity in the same range as the redocked co-crystallized ligand AT2_A (**Fig 7C**). The pose covers the binding site sufficiently and is stabilized by five hydrogen bonds (H-bonds); two with the binding site residues of the protein structure (ARG 381 and GLN 263) and another three with the COOH group of the heme cofactor (**Fig 7B**). While one of the OMe groups points towards the heme cofactor, the oxygen is in an unfavourable position to coordinate the iron. Note that the co-crystallized ligand AT2_A does not interact with the iron either, but its pyridine ring interacts with the nearby TYR 373 residue (H-bond). In contrast, compound C1-*R* obtained lower estimated affinities (in the μM range) and the predicted pose formed fewer hydrogen bonds. The good physicochemical and structural fit confirms that C1-*S* could be a potential ligand for iNOS.

### Mass spectroscopy identifies iNOS as a potential target for C1-*S*

We used mass spectrometry to determine potential, target and off-target, binding partners of C1-*S* and C1-*R* on microglia with and without LPS stimulation. We added a biotinylated linker to each compound to allow isolation from cell lysates and subjected the resulting protein mixture to mass spectrometry. We first determined that C1-*S* inhibits LPS-induced NO- release with an IC50 of 2.36 μM (data not shown).

**Fig 8:**
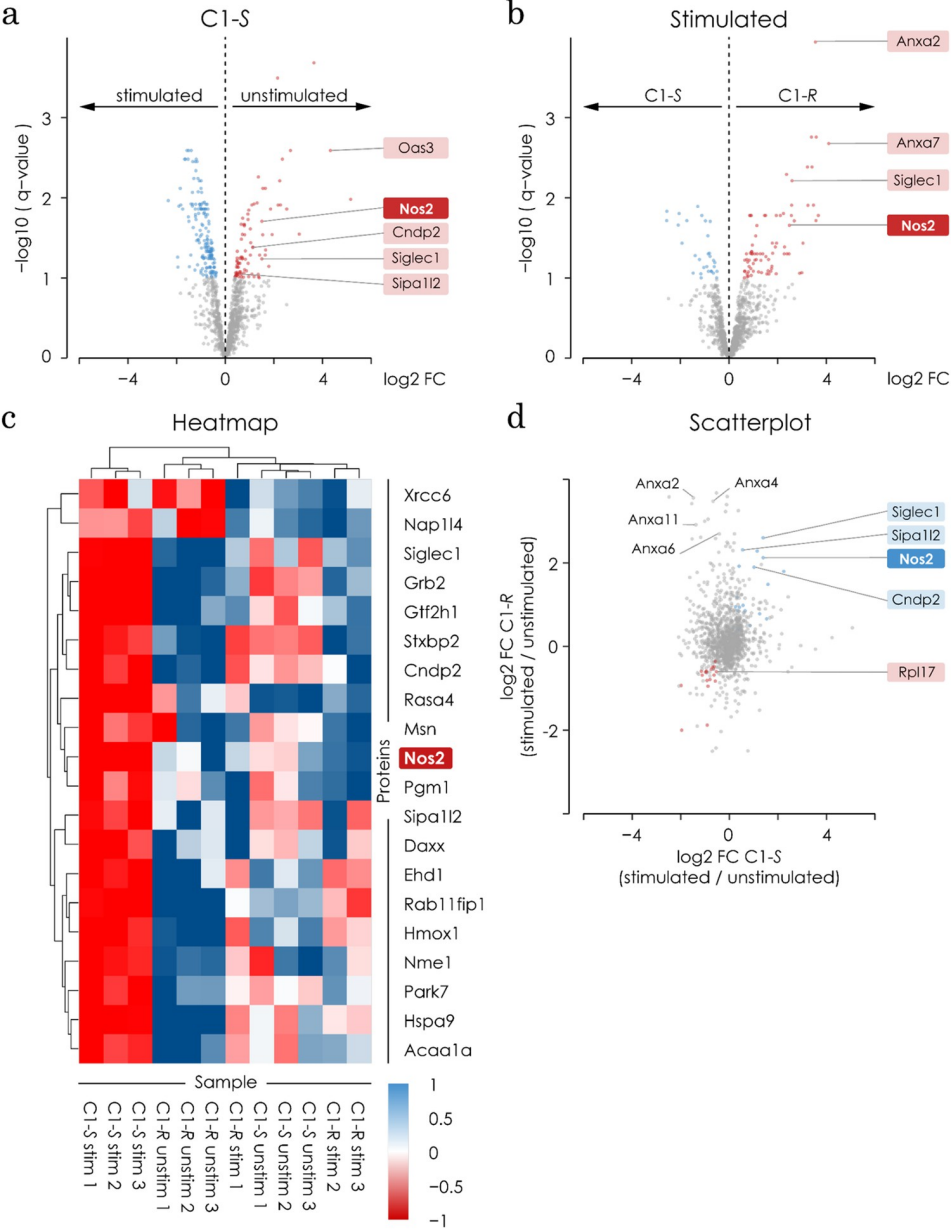
Target finding strategy using mass spectroscopy reveals NOS2 as a potential candidate. **a)** Volcano plot shows the distribution of all proteins comparing C1-S stimulated with LPS vs. C1-S unstimulated. All blue dots represent proteins that are significantly higher present in the unstimulated samples and lower in the stimulated samples and hence they have a negative log fold change. All orange dots represent proteins that are significantly higher present in the unstimulated group and hence they show a positive log fold change. NOS2 is amongst the proteins with a higher abundance in C1-S stimulated sample. Results are shown as the significance versus the magnitude of fold-changes. The analysis was made with a t-test (two-tailed *p<0.05). **b)** Volcano plot shows the distribution of all proteins comparing C1-R and C1-S stimulated both with LPS. The blue dots show the protein abundance in C1-R with a negative fold change (lower in C1-S) and the orange dots show the proteins present significantly higher in C1-S. NOS2 is amongst the proteins with a higher abundance in the C1-S sample. Results are shown as the significance versus the magnitude of fold-changes. The analysis was made with a t-test (two-tailed *p<0.05). **c)** Heatmap shows the top 20 proteins differently regulated between conditions. All proteins shown have the highest abundance in the C1-S stimulated condition (orange). NOS2 is amongst the top 20 proteins. **d)** Scatter plot comparing the proteins that are significantly enriched in the comparison C1-S stimulated versus unstimulated (x-Axis) with the proteins in the comparison C1-R stimulated versus unstimulated (y-axis). NOS2 is amongst the proteins enriched in the C1-S stimulated condition.

BV-2 cells were pre-treated for 1 hour with 10 μM C1-*S* or C1-*R* and subsequently stimulated with or without 1 μg/mL LPS for additional 48 hours. Values were imputed by an artificial replenishment following the Gaussian distribution [58], thus assuring that all proteins measured will be depicted in our results. The imputed data show a normal distribution across all lysates. All log2 ratios lie between 28 and 30, therefore, there was no need to normalize the values for further calculations. Since C1-*R* does not inhibit NO release, but has the same structure as C1-*S*, we hypothesised that proteins bound to C1-*S* but not to C1-*R* would be good target candidates unlike proteins binding to the compounds without stimulation. *NOS2* (the gene name for iNOS) shows up as a candidate significantly enriched in C1-*S* stimulated cells (**Fig 8A**). The scatter plot (**Fig 8B)** compares directly the proteins from the stimulated vs. unstimulated C1-*S* and C1-*R*. Also here, *NOS2* is amongst the genes enriched in the C1-*S* stimulated group. The heat map in **Fig 8C** shows the comparison of C1-*S* stimulated to all control samples (C1-*S* unstimulated, C1-*R* stimulated, C1-*R* unstimulated), where we would expect C1-*S*- specific targets to appear. Indeed, amongst others *NOS2* is recognized as a good target candidate. Together with the *in-silico* analysis, these results support our hypothesis that *NOS2* is a potential target candidate for C1-*S*. However, we here do not provide in vivo or in vitro information about the specificity of C1-*S* on iNOS over the other NOS species.

## Discussion

Here, we introduce a novel compound targeting the NO release by microglia. NO plays an important role as a cellular signalling molecule involved in the cardiovascular system regulating the blood pressure, in the CNS for retrograde transmission of signals along the synapses, and in the immune system defending against infiltrating bacteria and parasites. It has been shown that NO is involved in many different physiological and pathophysiological processes including diabetes, memory and learning, bowel movement, stroke, and differentiation of immune cells [9, 10]. The impact of NO is locally restricted and originates from cells, which express the distinct NOS isoforms, eNOS by endothelial cells, nNOS by neurons, and iNOS by cells of the immune system and activated microglia. The three NOS isoforms are regulated differently and have different catalytic properties and inhibitor sensitivities [59]. Inducible NO synthase (iNOS) is expressed by macrophages as a response of a pathologic stimulus and is needed for host defense against pathogens and for immune regulation [60]. *iNOS* is not expressed in the healthy brain but upregulated in microglia and astrocytes under pathological conditions [61]. Upon induction, iNOS is upregulated for several days and synthesizes NO, reaching concentrations that are toxic for neurons and other brain cells [62]. Because of the high concentrations of NO produced by iNOS in the brain under pathological conditions, its modulation is of great impact, making it a very interesting target for therapies for several CNS diseases [24].

In a large screen of small molecule compounds, we identified C1, as an attenuator of NO release in microglia and macrophages in a dose dependent manner with an IC_50_ value of 252 nM. C1 exists in four stereoisomers. The isomer C1-*S* has an IC50 of only 104 nM, thus having an even higher sensitivity than the C1 mix. The most common used NOS inhibitors L-NAME, a non-selective NOS inhibitor, and 1400W, an iNOS-selective inhibitor, have IC_50_ values of 13.5 μM and 0.2 μM respectively, measured in an enzyme-activity assay [63] while our data were determined in a cell-based assay. In a recent cell-based assay using the microglial cell line BV-2, two novel iNOS inhibitors had an IC_50_ of 1.229 μM (CM292) and 0.056 μM (CM544) [28]. This highlights the potency of our compound to target NO at a lower IC_50_ than most of the currently used compounds.

C1 has a unique chemical scaffold that distinguishes it from other NOS inhibitors. Most nonselective NOS inhibitors, like L-NMMA, or L-NAME, mimic the NOS substrate L-arginine antagonizing the binding to the highly conserved catalytic site of NOS [64] and thus potently inhibit all NOS isoforms [65]. Inhibitors more selective for iNOS, like 1400W or GW273629 and GW274150, show a larger structural diversity but bear the same amidine based functionality, which mimics the side chain of arginine and thus sharing a common binding motif. Our compound C1 has a unique peptide-like structure based on two amino acids proline and phenylglycine. In contrast to the inhibitors named before, C1 does not possess a structure resembling L-arginine or any highly polar amino acid head group nor any guanidine or amidine based functional groups, hinting towards a possible new mode of action in binding to iNOS.

We could show that C1 is able to reduce the NO release induced by three different pro-inflammatory stimuli, namely LPS, PolyIC, and IFNγ in a dose dependent manner in microglia and macrophages. The applied doses of C1 showed similar results for the different stimuli and cell types, with a minor effect on the NO reduction at a concentration of 25 nM and an almost complete blockage of NO release at a concentration of 2.5 μM. The analogous reduction in NO release independent from stimulus, presumes that C1 does not interfere with the pro-inflammatory pathways but acts downstream of those.

Other microglial cell functions such as the LPS-induced release of the pro-inflammatory cytokines IL1β, IL6 and TNFα, the migration towards ATP, and phagocytosis activity were not affected by C1. Showing that the complex regulation of cytokine release, migration and phagocytosis remain unchanged, we conclude that C1 does not interfere with the pro-inflammatory regulation underlining the assumption that C1 acts downstream of the pro-inflammatory pathways.

In microglia and macrophages, NO is almost exclusively produced by iNOS [11]. In contrast to eNOS and nNOS, the synthesis of iNOS is regulated by the transcription factors NFƙB, IRF1, and HIF1 [66]. We here could show that our compound does not interfere with the transcriptional regulation of *iNOS* since RNA levels were not affected. Moreover, we performed an NFƙB shift assay where C1 had no impact on NFƙB translocation into the nucleus (data not shown). Together, this indicates that C1 acts on a post-transcriptional level most likely by interfering with the enzymatic activity of the iNOS protein. Moreover, the reduction of NO release by microglia pre-stimulated with LPS also supports the regulation of iNOS on a post-transcriptional level. In an exemplary *in-silico* structure-based docking calculation, we showed that the isomer C1-*S* could potentially bind to iNOS and interact favorably with the enzyme-binding site. By using compounds with a biotinylated linker and a subsequent pull down of bound proteins that were analysed by mass spectroscopy we found several possible targets of the C1-*S* compound. Amongst other interesting candidate targets, we identified the protein *NOS2* (iNOS) in all downstream analysis. To exclude other proteins as potential targets and exclusively presenting *NOS2* as the only target, more in depth target deconvolution (3-Hybrid, phage display) assays and most probably refinements of the structure are needed, which is beyond the scope of this current study.

Selective inhibition of iNOS seems to be a promising target for therapy [24]. Aminoguanidine has been shown to act as an iNOS inhibitor and to be neuroprotective in the onset of stroke, acting in addition on other targets by inhibiting production of neurotoxins [67]. In experimental models of stroke, this drug can reduce the lesion volume in a dose dependent manner, acting even when administered 24h after the ischemic event [25, 68–70]. However, this drug has been refrained from clinical trials due to safety reasons [71]. Other compounds have also been tested, such as 1400W, which has been shown to decrease lesion volume and neurological deficits after stroke in rats [72] but safety questions have also risen. GW274150, GW273629 [73] show less toxicity but failed to show effectiveness for CNS diseases.

We have tested C1 in a pharmacokinetic study *in vivo*, which showed that mice tolerated the application of the substance without any obvious side effects. We have also shown that the compound can pass the blood brain barrier and can reach concentrations in the brain that exceed its IC_50_ for iNOS inhibition. Even at higher doses, C1 does not affect the viability, metabolic activity, proliferation and cell death of microglia and other brain cells like oligodendrocytes and astrocytes. We furthermore show that C1 can attenuate neurological symptoms after stroke. In our experimental stroke model, C1 improved motor skills, reduced laterality, and thus qualifies for further pre-clinical studies both in stroke and in other CNS conditions. A patent has been filed under the number 20192972.6–1109 (European Patent Office) and more work regarding the target, the specificity and the therapeutic potential of C1-*S* will be conducted in future studies. In order to test the safety and efficacy of the novel compound, the specificity of C1-*S* for iNOS over nNOS and eNOS needs to be shown in further ADME and PK studies.

## Supporting information

S1 File (PDF)

S1 Table (XLS)

S1 Fig C1 does not change metabolic activity or scavenges NO from cultured microglia. **a)** The metabolic activity of primary cultured neonatal microglia was measured using an AlamarBlue assay. DMSO alone showed no impact on the metabolic activity of the cells. 20 μM of C1 reduced the metabolic activity to 84% ± 3.99%, however not below the unstimulated control (69% ± 2.47%, white bar). **b)** C1 and DMSO do not scavenge NO or independently impact the Griess assay. NO enriched supernatant taken from LPS stimulated microglia (1 μg/mL LPS for 48 hours) was incubated with C1 in DMSO or DMSO alone (concentration range is the same as in **a)** for 24 hours. Treatment with C1 or DMSO showed no dose dependent reduction on the NO concentration in the supernatant when compared to the untreated supernatant.
(PNG)

S2 Fig C1 alone does not evoke NO release, an increase in cell activity or release of pro-inflammatory cytokines. **a)** The effect of C1 on the NO release in unstimulated microglia showed no significant difference. Microglia were treated with C1 (0.025 μM, 0.25 μM, or 2.5 μM, in red) or its corresponding concentration of DMSO (1.25x10^-5^, 12.5x10^-5^, or 125x10^-5^ v/v, in grey) for 48 hours. Untreated microglia stimulated with 1 μg/mL LPS for 48 hours were set as positive control (in black). All values were normalised to the positive control. As shown in **Fig 1**, LPS stimulation induced a significant increase in NO release (p<0.0001). Treatment with C1 or DMSO did not show any significant difference compared to untreated microglia (in white). **b)** Using the same protocol as in **a**, the metabolic activity was assessed using AlamarBlue assay. LPS stimulation induced a significant increase in metabolic activity (in black) compared to the unstimulated microglia (in white) (p<0.0001). Treatment with C1 (in blue) or DMSO (in grey) did not show any significant difference compared to untreated microglia. **c)** Treatment with C1 (in blue) did not induce any significant changes in the release of pro-inflammatory cytokines (left: IL1β, middle: IL6, right: TNFα). Using the same protocol as described in **Fig 2** the release of IL1β, IL6, and TNFα was measured using ELISA. Untreated microglia stimulated with 1 μg/mL LPS for 48 hours were set as positive control (in black). All values were normalised to the positive control respectively. As shown in **Fig 2** LPS stimulation induced a significant increase in the release of IL1β, IL6, and TNFα (all p<0.0001). Treatment with C1 (in blue) or DMSO (in grey) did not show any significant difference compared to untreated microglia in all measured cytokines. *** p<0.001 comparing to plain medium control (1way ANOVA followed by Bonferroni’s post-hoc test).
(PNG)

S3 Fig C1 shows similar results on macrophages as for microglia and does not interfere with cell activity, proliferation, and cell death are not affected in non-stimulated conditions. **a)** Adult bone marrow derived macrophages were isolated and pre-treated for 1 hour with C1 (0.025 μM, 0.25 μM, or 2.5 μM, in red) or its corresponding concentration of DMSO (1.25x10^-5^, 12.5x10^-5^, or 125x10^-5^ v/v, in grey), followed by 48 hours stimulation with 1 μg/mL LPS. In addition, untreated macrophages were stimulated 48 hours with 1 μg/mL LPS (in black) or kept in plain medium (negative control in white). 0.25 μM C1 reduced the NO release significantly compared to positive control (p < 0.0001) and DMSO (p < 0.0001) and 2.5 μM C1 reduced the NO release to similar level as the negative control (C1: 4.85% ± 0.65 SEM, plain medium: 6.01% ± 0.62 SEM, p > 0.9999) significantly different to the positive control (p < 0.0001) and DMSO (p < 0.0001). **b)** The same stimulation protocol as in **a)** was used, replacing the LPS by 100 μg/mL PolyIC for 24 hours and the NO release was measured. Similar as for the LPS stimulation, 0.25 μM showed a significant decrease in NO concentration compared to positive control (p < 0.0001) and DMSO (p < 0.0001). 2.5 μM of C1 lead to a decrease (15.01±10.32%, p = 0.9751) to the same NO concentration as induced by the negative control (24.30±12.91%), which is significantly different from the positive control (p < 0.0001) and DMSO (p < 0.0001). **c)** When the stimulus of the protocol, used in **a**, was exchanged by 100 ng/mL IFNγ (48 hours stimulation), C1 reduced the NO release in a dose dependent manner, reaching the same level as the negative control when using 2.5 μM C1 (control 29% versus treated 18%, p = 0.1253) and DMSO reduced the IFNγ induced NO release in these cells independently of the applied dose (1.25x10^-5^ v/v: 85%, p<0.0043; 12.5x10^-5^ v/v: 83%, p<0.0016; 125x10^-5^ v/v: 85%, p<0.0046), similar to the effect observed in microglia when stimulated with IFNγ. **d)** When stimulated with 1 μg/mL LPS (for 48 hours, using the same protocol as referred above C1 caused a dose independent decrease in cell activity for all 3 concentrations of C1 (0.025μM: 78.47 ± 31.61%, p = 0.0245; 0.25μM: 69.31 ± 36.50%, p = 0.001; 2.5μM: 66.48 ± 31.17%, p<0.001) in comparison to the positive control (100 ± 4.74%) and when cells were treated with 2.5 μM C1 there was a reduction of the cell activity also when compared to the DMSO control (p = 0.0310). Treatment with DMSO did not show a significant decrease. **e)** When stimulated with 100 μg/mL PolyIC for 24 hours using the same protocol as referred above the cells undergo a reduction of metabolic activity after treatment with 0.025 μM C1 compared to DMSO stimulation (p = 0.0053) and the positive control (p = 0.0138). Higher concentrations of C1 did not show a significant difference. **f)** When stimulated with IFNγ for 48 hours using the same protocol as referred above the cells undergo a significant decrease in metabolic activity after treatment with 0.25 μM (p = 0.0283) and 2.5 μM (p = 0.0357) compared to the positive control, but not their innate DMSO control. **g)** The pro-inflammatory cytokines IL1β, IL6, and TNFα, were measured after a 48 hours LPS (1 μg/mL) stimulation. The protocol was performed as noted in **a**. The LPS induced release of IL1β and IL6 was not changed in the presence of C1 or DMSO. DMSO showed a significant effect on the release of TNFα in the concentrations 1.25x10^-5^ v/v (p < 0.0001) and 12.5x10^-5^ v/v (p = 0.005). 2.5 μM C1 decreased the release of TNFα significantly to 91% compared to the positive control (p = 0.0313). *** p<0.001 ** p<0.01 * p<0.05 comparing to stimulated control, ### p<0.001 ## p<0.01 ## p<0.05 comparing to DMSO (1way ANOVA followed by Bonferroni’s post-hoc test).
(PNG)

S4 Fig C1 has no negative effect on brain cells. **a)** The metabolic activity of primary cultured neonatal microglia, primary cultured neonatal astrocytes and the oligodendrocyte cell line OLN-93 was evaluated under physiological condition (unstimulated). The cells were treated for 48 hours with 2.5 μM C1 (in blue), or its corresponding concentration of DMSO (125x10^-5^ v/v, in grey) or plain medium (in white). DMSO (p = 0.0439) and C1 (p = 0.0402) showed a significant increase in metabolic activity on microglia compared to plain medium. Comparing DMSO and C1 showed no significant difference. DMSO and C1 showed no influence on astrocytes. C1 increased the metabolic activity in oligodendrocytes compared to DMSO (p = 0.0431) but not compared to plain medium (p = 0.2140). **b)** Using the same experimental setup as in **Fig 3**, the proliferation and cell death was measured using a propidium iodide based assy. C1 increased the proliferation in microglia significantly compared to plain medium (p = 0.0154) and DMSO (p < 0.0001), but did not show an effect on the other tested cell types. DMSO did increase the proliferation of astrocytes compared to plain medium (p = 0.0060). **c)** DMSO increased the percentage of dead cells in microglia significantly compared to plain medium (p = 0.0096). Treatment with C1 kept the percentage of dead cells similar to plain medium (C1: 8.49%, plain medium: 8.26%) with no significant difference. However, it showed a significant reduction from the elevated DMSO level (p = 0.0201). ** p<0.01 * p<0.05 comparing to stimulated control, ^###^ p<0.001 ^##^ p<0.01 ^#^ p<0.05 compared to DMSO (1way ANOVA followed by Bonferroni’s post-hoc test).
(PNG)
