# Supplementary material for: Small molecule inhibiting microglial nitric oxide release could become a potential treatment for neuroinflammation
Source: PLoS One. 2023 Feb 6;18(2):e0278325. doi: 10.1371/journal.pone.0278325 (PMC9901772; doi:10.1371/journal.pone.0278325)
Supplement: S1 File — (PDF) [file pone.0278325.s001.pdf]

Supplemental material - Synthesis - description and raw data

All raw data are deposited in Dryad DOI https://doi.org/10.5061/dryad.q83bk3jms. We here describe the details of the synthesis. Reagents and solvents were obtained from commercial suppliers and used without further purification. Organic solvents were dried using molecular sieves. ^{1}H-NMR and ^{13}C-NMR spectra were recorded either on AV 300 MHz or on AV 600 MHz from Bruker. Chemical shifts are recorded in parts per million (ppm,   ). Spin multiplicities are described as s (singlet), d (duplet), t (triplet), q (quartet) and m (mulitplet). Coupling constant (*J*) are recorded in Hz. NMR data were analyzed with MestReNova software. Mass analyses were performed with two different spectrometers using the same column. LCMS (method 1): Instrument: Agilent Technologies 6230 Accurate Mass TOF LC/MS linked to Agilent Technologies HPLC 1260 Series; Column: Thermo Accuore RP-MS; Particle Size: 2.6 µM Dimension: 30 x 2.1 mm; Eluent A: H_2O with 0.1 % TFA Eluent B: MeCN with 0.1 % TFA; Gradient: 0.00 min 95 % A, 0.2 min 95% A, 1.1 min 1% A, 2.5 min Stoptime, 1.3 min Posttime; Flow rate: 0.8 ml/min; UV-detection: 220 nm, 254 nm, 300 nm. LCMS (method 2): Instrument: Agilent Technologies 6120 Quadrupole LC/MS linked to Agilent Technologies HPLC 1290 Infinity; Column: Thermo Accuore RP-MS; Particle Size: 2.6 µM Dimension: 30 x 2.1 mm; Eluent A: H_2O with 0.1 % TFA Eluent B: MeCN with 0.1 % TFA; Gradient: 0.00 min 95 % A, 0.2 min 95% A, 1.1 min 1% A, 2.5 min Stoptime, 1,3 min Posttime; Flow rate: 0.8 ml/min; UV-detection: 220 nm, 254 nm, 300 nm. Purification of the compounds by chromatography was obtained using a CombiFlash Rf 200 UV-VIS System from Axel Semrau®.

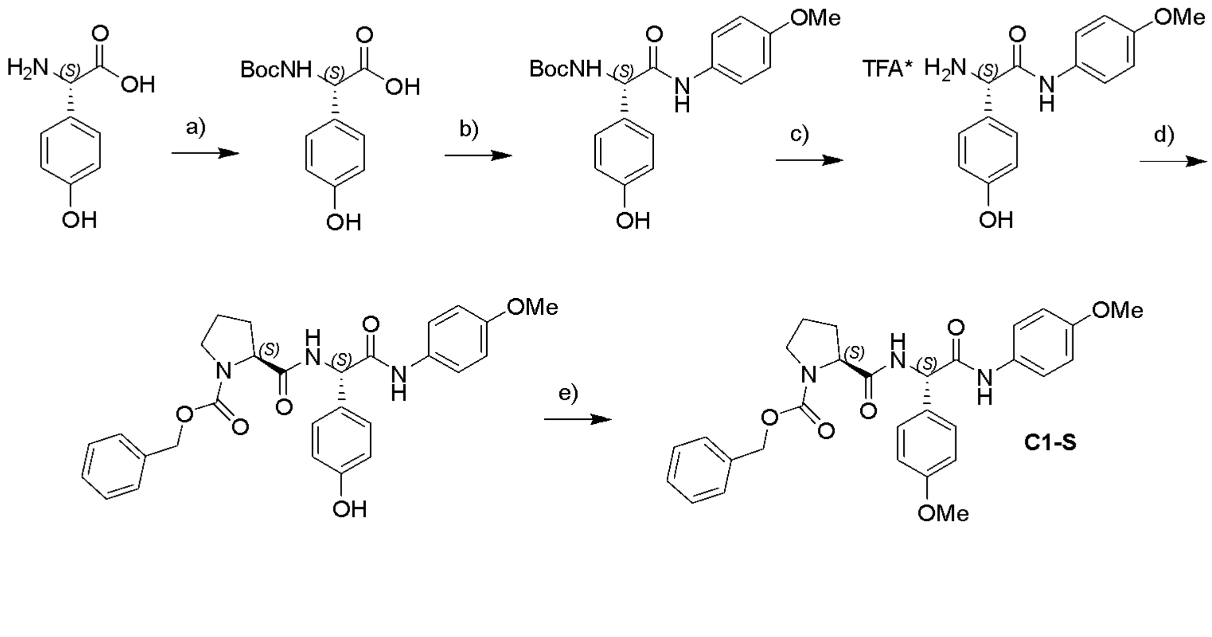

Figure 1 supporting information: 5 step synthesis of **C1-S**: a) Boc_2O, dioxane/H_2O, quant. b) p-methoxy-aniline, HATU, HOBt, 1 eq. DIPEA, 78 %, c) TFA, DCM, quant. d) Cbz-(S)-Pro-OH, HATU, HOBt, DIPEA, 75 %, e) MeI, DMF, K_2CO_3, 67 %.

The synthesis started with the Boc-protection of the (S)-enantiomer of 4-Hydroxyphenylglycine, followed by amidation with *para*-methoxy-aniline under standard peptide coupling conditions using 1 eq. DIPEA in order to minimize the risk of racemization to give **2** and **6** and in 78 % yield. Deprotection of the Boc-group was accomplished with TFA in DCM. The amine was coupled to (S)-Cbz-Pro-OH under standard peptide coupling conditions and subsequently purified by chromatography resulting in 75 % yield. The last step was the methylation of the Hydroxy-group using MeI in DMF under basic conditions. The same procedure was applied to the (R)-enantiomer of 4-Hydroxyphenylglyine with similar yields. In both cases no racemization of the stereocenters were observed.

(S)-2-((*tert*-Butoxycarbonyl)amino)-2-(4-hydroxyphenyl)acetic acid (**1**)

2 g (12 mmol) (S)-4-Hydroxy-phenylglycine were dissolved in dioxane/H_2O (1:1) and 3.13 g (14 mmol) of Boc_2O was added in one portion at RT. The mixture was stirred for 4 h at RT. Dioxane was removed under reduced pressure and the resulting aqueous solution was extracted EtOAc. The organic extracts were combined and dried over Na_2SO_4. After removal of the solvent 3.1 g (12 mmol) of the product was obtained as white powder in quantitative yield.

^{1}H NMR (300 MHz, DMSO-d_6) δ 7.40 (d, *J* = 8.2 Hz, 1H), 7.18 (d, *J* = 8.7 Hz, 2H), 6.71 (d, *J* = 8.6 Hz, 2H), 4.95 (d, *J* = 8.1 Hz, 1H), 1.38 (s, 9H). ^{13}C NMR (75 MHz, DMSO-d_6) δ 173.2, 157.5, 155.6, 129.4, 128.0, 115.5, 78.7, 60.2, 57.5, 28.7. LCMS: R_t = 1.40 min; HRMS (ESIpos): m/z $[M+Na]^+$ calcd for $C_{13}H_{17}NO_5$ 290.0999 found 290.1000.

tert-Butyl-(S)-(1-(4-hydroxyphenyl)-2-((4-methoxyphenyl)amino)-2-oxoethyl)carbamate (**2**)

2.1 g (7.9 mmol) of **1** were dissolved in DMF. 986 mg (7.9 mmol) p-anisidine, 1.2 g (7.9 mmol) HOBt, 2.99 g (7.9 mmol) HATU and 1.37 ml (7.9 mmol, 1 eq) DIPEA were added at RT. The mixture was stirred for 4 h at RT. DMF was evaporated and the mixture was dissolved in EtOAc. The organic solution was extracted with 1N HCl solution to remove DIPEA and HOBt. The organic layer was dried over Na_2SO_4 and the crude product was purified by chromatography on silica gel eluting with a gradient of DCM/MeOH. The fractions containing the product were combined and the solvent evaporated under reduced pressure. Yield: 2.3 g (78%).

^{1}H NMR (300 MHz, DMSO-d_6) δ 10.00 (s, 1H), 9.43 (s, 1H), 7.48 (d, *J* = 9.1 Hz, 2H), 7.27 (d, *J* = 8.6 Hz, 2H), 6.87 (d, *J* = 9.2 Hz, 2H), 6.72 (d, *J* = 8.6 Hz, 2H), 5.19 (d, *J* = 8.2 Hz, 1H), 3.70 (s, 3H), 1.39 (s, 9H). ^{13}C NMR (75 MHz, DMSO-d_6) δ 169.5, 157.4, 155.7, 155.5, 132.5, 129.0, 121.0, 115.5, 114.3, 78.7, 58.3, 55.6, 28.7. LCMS: R_t = 1.67 min; HRMS (ESIpos): m/z $[M+H]^+$ calcd for $C_{13}H_{17}NO_5$ 373.1758 found 373.1555.

(S)-2-Amino-2-(4-hydroxyphenyl)-N-(4-methoxyphenyl)acetamide (**3**)

2.3 g (6.1 mmol) of **2** were dissolved in 100 ml DCM/TFA mixture (9:1) and stirred at RT for 1 h. The solvent was removed under reduced pressure and EE/hexane was added several times and iteratively removed under reduced pressure until a white foam was contained. Yield: 2.6 g (quant.)

^{1}H NMR (300 MHz, DMSO-d_6) δ 10.46 (s, 1H), 8.63 (s, 2H), 7.49 (d, *J* = 9.2 Hz, 2H), 7.37 (d, *J* = 8.8 Hz, 2H), 6.91 (d, *J* = 9.2 Hz, 2H), 6.83 (d, *J* = 8.7 Hz, 2H), 4.96 (s, 1H). ^{13}C NMR (75 MHz, DMSO-d_6) δ 166.3, 158.8, 156.2, 131.6, 129.8, 124.3, 121.3, 116.0, 114.5, 56.2, 55.6. LCMS: R_t = 0.58 min; HRMS (ESIpos): m/z $[M+H]^+$ calcd for $C_{15}H_{16}N_2O_3$ 273.1234 found 273.1234.

Benzyl-(S)-2-(((S)-1-(4-hydroxyphenyl)-2-((4-methoxyphenyl)amino)-2-oxoethyl)-carbamoyl)-pyrrolidine-1-carboxylate (**4**)

2.6 g (6.7 mmol) of **3** were dissolved in 60 ml DMF and 1.68 g (6.7 mmol) Cbz-(S)-Pro-OH, 1.0 g (6.7 mmol) HOBt, 2.56 g (6.7 mmol) HATU and 2.3 ml (13.5 mmol) DIPEA were added. The mixture was stirred for 1h and the solvent was removed under reduced pressure. The mixture was dissolved in EtOAc and extracted with 1N HCl. The organic layer was dried with Na_2SO_4 and removed under reduced pressure. The crude product was purified by chromatography on silica gel eluting with a gradient of DCM/MeOH. The fractions containing the product were combined and the solvent evaporated under reduced pressure. Yield: 2.55 g (75%).

^{1}H-NMR (300 MHz, DMSO-d_6) δ cis/trans (10.11 (s, 1H) and 10.05 (s, 1H)), cis/trans (9.48 (s, 1H) and 9.45 (s, 1H)), cis/trans (8.57 (d, *J* = 7.4 Hz, 1H) and 8.50 (d, *J* = 7.3 Hz, 1H)), 7.49 (d, *J* = 8.5 Hz, 2H), 7.41 - 7.33 (m, 2H), 7.32 - 7.18 (m, 5H), 6.87 (d, *J* = 8.5 Hz, 2H), 6.79 - 6.66 (m, 2H), cis/trans (5.45 (d, *J* = 7.3 Hz, 1H) and 5.41 (d, *J* = 7.4 Hz, 1H)), cis/trans (5.07 (s, 2H) and 5.00 - 4.89 (m, 2H)), cis/trans (4.47 (dd, *J* = 8.7, 3.5 Hz, 1H) and 4.40 (dd, *J* = 8.7, 3.1 Hz, 1H)), 3.71 (s, 3H), 3.50 - 3.38 (m, 2H), 2.22 - 2.05 (m, 1H), 1.95 - 1.73 (m, 3H). ^{13}C-NMR (75 MHz, DMSO-d_6): cis/trans (172.4 and 172.0), 169.1, 157.5, 155.7, cis/trans (137.5 and 137.4), 132.5, 129.1, 129.0, 128.9, cis/trans (128.6 and 128.5), 128.2, 127.9, 127.4, 120.9, 115.6, 114.3, cis/trans (66.3 and 66.2), cis/trans (59.8 and 59.1), cis/trans (57.0 and 56.9), 55.6, cis/trans (47.6 and 47.0), cis/trans (31.7 and 30.5), cis/trans (24.4 and 23.5). LCMS: R_t = 1.71 min; HRMS (ESIpos): m/z $[M+Na]^+$ calcd for $C_{28}H_{29}N_3O_6$ 526.1942 found 526.1949.

Benzyl-(S)-2-(((S)-(4-methoxyphenylcarbamoyl)(4-methoxyphenyl)methylcarbamoyl)pyrrolidine-1-carboxylate (**C1-S**).

200 mg (0.4 mmol) of **4** were dissolved in 4 ml DMF. 110 mg K_2CO_3 (0.8 mmol) and 25 µl (0.4 mmol) MeI were added. The mixture was stirred for 16 h at RT. K_2CO_3 was filtered off, the solvent and MeI were removed under reduced pressure and the crude product was purified by chromatography on silica gel eluting with a gradient of Hex/EE. The fractions containing the product were combined and the solvent evaporated under reduced pressure. Yield: 140 mg (67%).

^{1}H-NMR (300 MHz, DMSO-d_6) δ cis/trans (10.11 (s, 1H) and 10.05 (s, 1H)), cis/trans (8.58 (d, J = 7.3 Hz, 1H) and 7.49 (d, J = 9.1 Hz, 1H)), 7.49 (d, J = 8.9 Hz, 2H), 7.45 - 7.31 (m, 4H), 7.28 - 7.21 (m, 3H), 6.94 (d, J = 8.9 Hz, 1H), 6.90 - 6.83 (m, 3H), cis/trans (5.52 (d, J = 7.6 Hz, 1H) and 5.48 (d, J = 7.6 Hz, 1H)), cis/trans (5.07 (s, 2H) and 5.00 - 4.89 (m, 2H), cis/trans (4.49 (dd, J = 8.5, 3.5 Hz, 1H) and 4.41 (dd, J = 8.6, 3.1 Hz, 1H)), 3.71 (s, 6H), 3. 3.49 - 3.36 (m, 2H), 2.27 - 2.04 (m, 1H), 1.94 - 1.73 (m, 3H). ^{13}C NMR (75 MHz, DMSO-d_6) δ cis/trans (172.4 and 172.1), cis/trans (168.9 and 168.9), cis/trans (159.3 and 159.3), 155.7, cis/trans (154.5 and 154.2), cis/trans (137.5 and 137.4), 132.4, cis/trans (130.4 and 130.3), 129.0, 128.9, 128.6, 128.2, 127.9, 127.3, 121.0, 114.3, 114.3, 114.2, cis/trans (66.3 and 66.1), cis/trans (59.8 and 59.2), 56.8, 56.7, cis/trans (55.6 and 55.6), cis/trans (47.6 and 47.0), cis/trans (31.8 and 30.5), cis/trans (24.4 and 23.5). LCMS: R_t = 1.91 min; HRMS (ESIpos): m/z $[M+H]^+$ calcd for $C_{29}H_{31}N_3O_6$ 518.2286 found 518.2288.

The same procedures were applied to synthesize the **C1-R**.

(R)-2-((*tert*-Butoxycarbonyl)amino)-2-(4-hydroxyphenyl)acetic acid (**5**)

LCMS: R_t = 0.98 min; MS (ESIpos) m/z = 290.1 $[M+Na]^+$.

tert-Butyl-(R)-(1-(4-hydroxyphenyl)-2-((4-methoxyphenyl)amino)-2-oxoethyl)carbamate (**6**)

^{1}H NMR (300 MHz, DMSO-d_6) δ 10.00 (s, 1H), 9.43 (s, 1H), 7.48 (d, J = 9.1 Hz, 2H), 7.27 (d, J = 8.7 Hz, 2H), 6.87 (d, J = 9.2 Hz, 2H), 6.72 (d, J = 8.6 Hz, 2H), 5.18 (d, J = 8.2 Hz, 1H), 3.70 (s, 3H), 1.39 (s, 9H). ^{13}C NMR (75 MHz, DMSO-d_6) δ 169.5, 157.4, 155.7, 155.5, 132.5, 129.2, 129.0, 121.0, 115.5, 114.3, 78.7, 58.3, 55.6, 28.6. LCMS: R_t = 1.16 min; MS (ESIpos) m/z = 395.5 $[M+Na]^+$.

(R)-2-Amino-2-(4-hydroxyphenyl)-N-(4-methoxyphenyl)acetamide (**7**)

^{1}H NMR (300 MHz, DMSO-d_6) δ 10.45 (s, 1H), 8.62 (s, 2H), 7.49 (d, J = 9.2 Hz, 2H), 7.37 (d, J = 8.8 Hz, 2H), 6.91 (d, J = 9.2 Hz, 2H), 6.83 (d, J = 8.7 Hz, 2H), 4.95 (s, 1H), 3.72 (s, 3H). ^{13}C NMR (75 MHz, DMSO-d_6) δ 166.3, 158.8, 156.2, 131.6, 129.8, 124.3, 121.3, 116.0, 114.5, 56.2, 55.6. LCMS: R_t = 0.84 min; MS (ESIpos) m/z = 273.1 $[M+H]^+$.

Benzyl-(S)-2-(((R)-1-(4-hydroxyphenyl)-2-((4-methoxyphenyl)amino)-2-oxoethyl)carbamoyl)-pyrrolidine-1-carboxylate (**8**)

^{1}H NMR (300 MHz, DMSO-d_6) δ cis/trans (10.22 (s, 1H) and 10.02 (s, 1H)), 9.45 (s, 1H), 8.73 - 8.65 (m, 1H), 7.57 - 7.48 (m, 2H), 7.40 - 7.32 (m, 2H), 7.30 - 7.08 (m, 5H), 6.89 (d, J = 9.2 Hz, 2H), 6.74 (d, J = 7.9 Hz, 2H), cis/trans (5.59 (d, J = 8.2 Hz, 1H) and 5.49 (d, J = 7.9 Hz, 1H)), cis/trans (5.08 (s, 2H) and

4.99 (s, 2H)), cis/trans (4.48 (dd, dd, J = 8.2, 3.8 Hz 1H) and 4.40 (dd, J = 8.2, 3.9 Hz, 1H)), 3.47 - 3.38 (m, 2H), 2.22 - 2.04 (m, 1H), 1.87 - 1.68 (m, 3H). ^{13}C NMR (75 MHz, DMSO-d_6) δ cis/trans (172.0 and 171.9), 168.8, 157.4, cis/trans (155.8 and 155.8), cis/trans (154.6 and 154.2), cis/trans (137.4 and 137.3), cis/trans (132.5 and 132.3), 129.5, 129.4, 128.9, 128.6, 128.4, 128.2, 127.9, 127.7, 127.4, cis/trans (121.2 and 121.0), 115.6, 114.3, cis/trans (66.4 and 66.3), cis/trans (60.2 and 60.0), cis/trans (56.6 and 56.3), 55.6, cis/trans (47.6 and 47.1), cis/trans (31.7 and 30.4), cis/trans (24.4 and 23.5).LCMS: R_t = 1.72 min; HRMS (ESIpos): m/z $[M+H]^+$ calcd for $C_{28}H_{29}N_3O_6$ 504.1929 found 504.1942.

Benzyl-(S)-2-(((R)-1-(4-methoxyphenyl)-2-((4-methoxyphenyl)amino)-2-oxoethyl)carbamoyl)-pyrrolidine-1-carboxylate (**C1-R**)

^{1}H NMR (300 MHz, DMSO-d_6) δ cis/trans (10.26 (s, 1H) and 10.08 (s, 1H)), cis/trans (8.78 (d, J = 8.4 Hz, 1H) and 8.75 (d, J = 7.9 Hz, 1H)), 7.56 - 7.47 (m, 2H), 7.42 - 7.34 (m, 4H), 7.31 - 7.08 (m, 3H), 6.95 - 6.86 (m, 4H), cis/trans (5.65 (d, J = 8.3 Hz, 1H) and 5.56 (d, J = 8.1 Hz, 1H)), cis/trans (5.09 (s, 2H) and 5.00 (s, 2H)), cis/trans (4.49 (dd, J = 8.6, 3.5 Hz, 1H) and 4.41 (dd, J = 8.6, 3.8 Hz, 1H)), 3.49 - 3.38 (m, 2H), 2.22 - 2.06 (m, 1H), 1.87 - 1.68 (m, 3H). ^{13}C NMR (75 MHz, DMSO-d_6) δ ^{13}C NMR (75 MHz, DMSO-d_6) δ cis/trans (172.1 and 171.9), 168.6, 159.2, cis/trans (155.9 and 155.8), cis/trans (154.6 and 154.2), cis/trans (137.4 and 137.3), cis/trans (132.4 and 132.2), cis/trans (131.3 and 131.2), 128.9, 128.6, 128.4, 128.2, 127.9, 127.7, 127.4, cis/trans (121.2 and 121.0), cis/trans (114.4 and 114.3), cis/trans (66.4 and 66.3), cis/trans (60.0 and 59.4), cis/trans (56.5 and 56.2), 55.6, 55.6, cis/trans (47.6 and 47.1), cis/trans (31.7and 30.5), cis/trans (24.4 and 23.5). LCMS: R_t = 1.86 min; HRMS (ESIpos): m/z $[M+H]^+$ calcd for $C_{29}H_{31}N_3O_6$ 518.2286 found 518.2288.

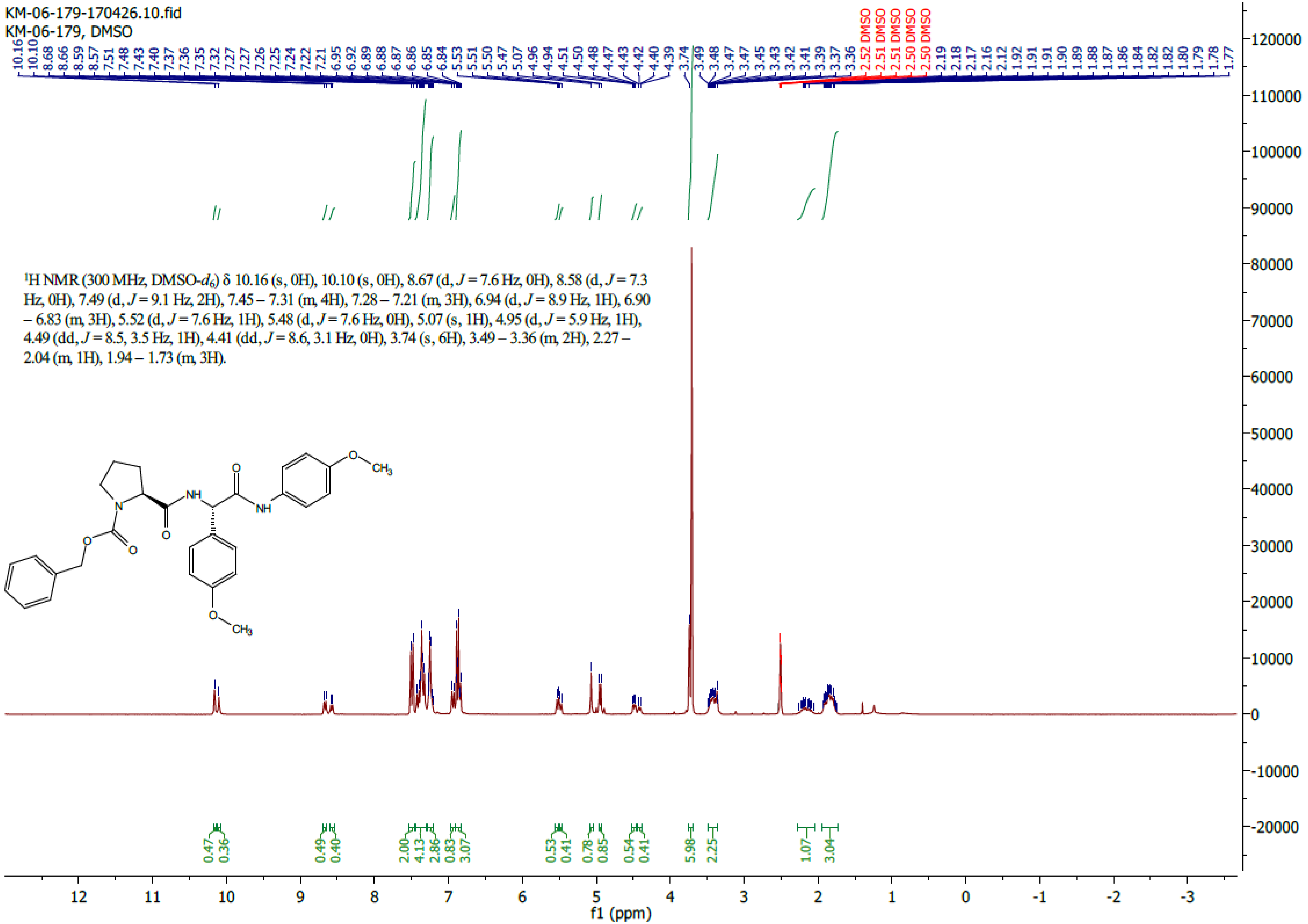

^{1}H NMR (300 MHz, DMSO-d_6) δ 10.16 (s, 0H), 10.10 (s, 0H), 8.67 (d, J = 7.6 Hz, 0H), 8.58 (d, J = 7.3 Hz, 0H), 7.49 (d, J = 9.1 Hz, 2H), 7.45 – 7.31 (m, 4H), 7.28 – 7.21 (m, 3H), 6.94 (d, J = 8.9 Hz, 1H), 6.90 – 6.83 (m, 3H), 5.52 (d, J = 7.6 Hz, 1H), 5.48 (d, J = 7.6 Hz, 0H), 5.07 (s, 1H), 4.95 (d, J = 5.9 Hz, 1H), 4.49 (dd, J = 8.5, 3.5 Hz, 1H), 4.41 (dd, J = 8.6, 3.1 Hz, 0H), 3.74 (s, 6H), 3.49 – 3.36 (m, 2H), 2.27 – 2.04 (m, 1H), 1.94 – 1.73 (m, 3H).

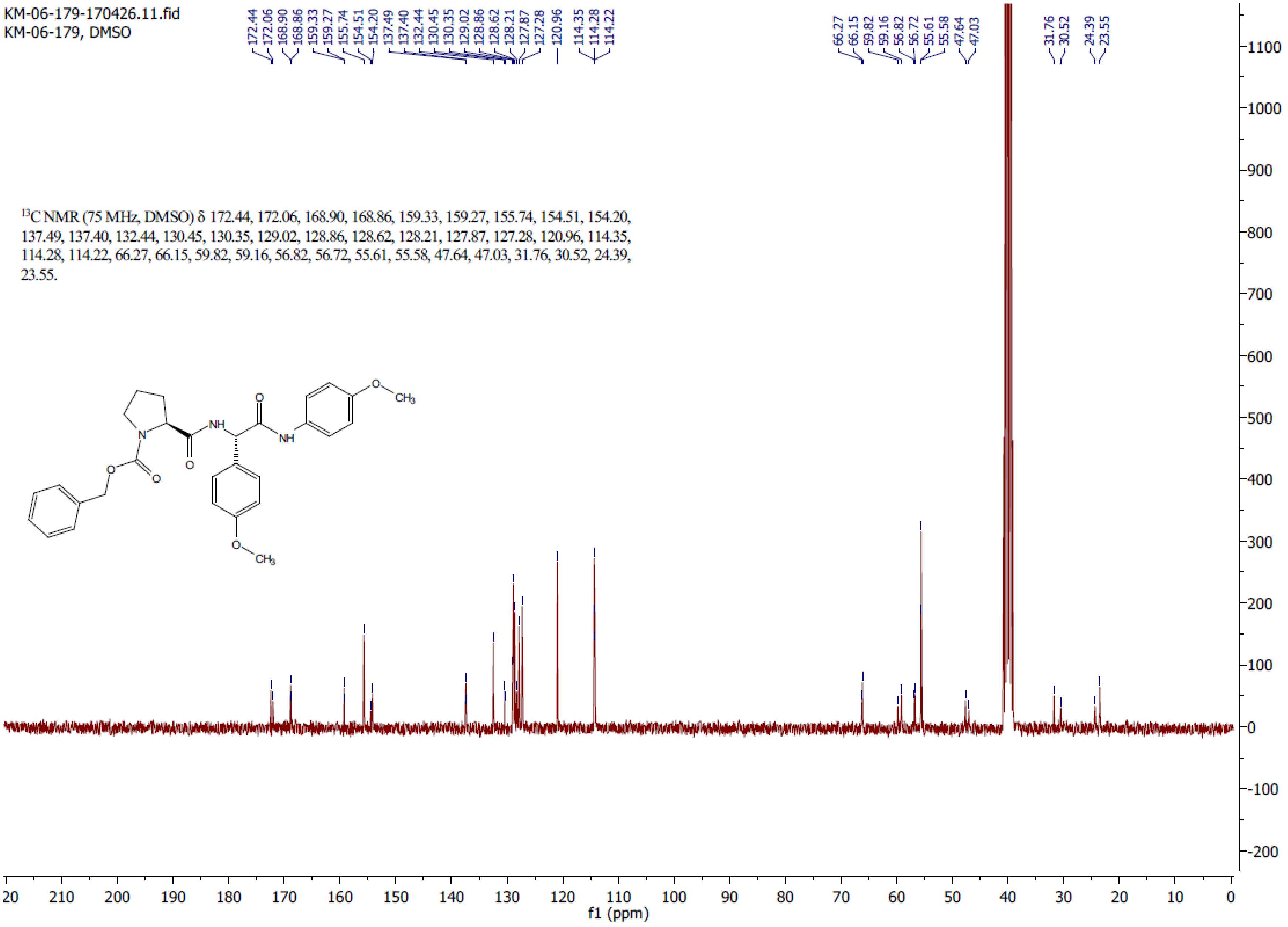

^{13}C NMR (75 MHz, DMSO) δ 172.44, 172.06, 168.90, 168.86, 159.33, 159.27, 155.74, 154.51, 154.20, 137.49, 137.40, 132.44, 130.45, 130.35, 129.02, 128.86, 128.62, 128.21, 127.87, 127.28, 120.96, 114.35, 114.28, 114.22, 66.27, 66.15, 59.82, 59.16, 56.82, 56.72, 55.61, 55.58, 47.64, 47.03, 31.76, 30.52, 24.39, 23.55.

Qualitative Compound Report

Fragmentor Voltage 150 **Collision Energy** 0 **Ionization Mode** ESI

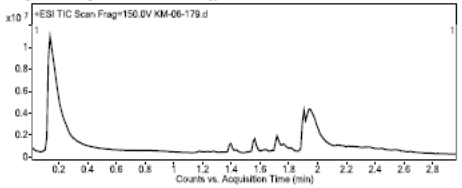

Fragmentor Voltage **Collision Energy** **Ionization Mode**

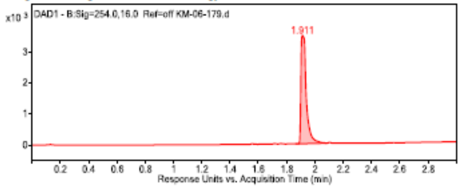

User Chromatogram Peak List

Compound Name	Compound Number	RT	Height	Height %	Area	Area %	Area Sum %	Width
Cpd 1: 1.937	1	1.911	3500.33	100	9275.18	100	100	0.222

Compound Table

Compound Label	RT	Mass	Abund	Formula	Tgt Mass	Diff (ppm)	Purity Value	Purity Result
Cpd 1: C29 H31 N3 O6	1.937	517.2219	542398	C29 H31 N3 O6	517.2213	1.12	100	Pass

Compound Label	*m/z*	RT	Algorithm	Mass
Cpd 1: C29 H31 N3 O6	518.2288	1.937	Find By Formula	517.2219

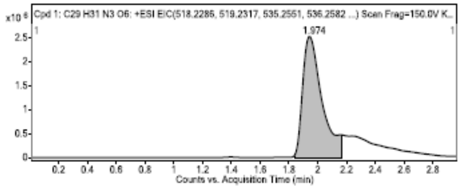

MS Spectrum

 Printed at: 1:53 PM on:5/8/2017

Qualitative Compound Report

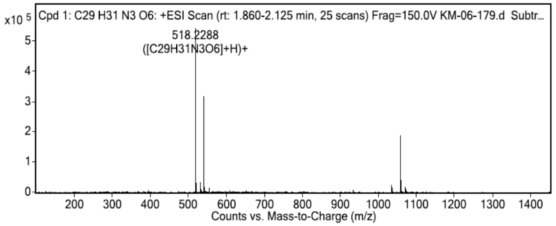

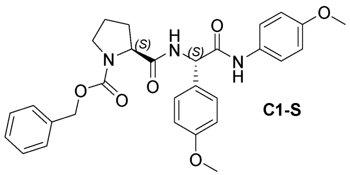

C1-S

Chemical Formula: $C_{29}H_{31}N_3O_6$
Exact Mass: 517,2213
Molecular Weight: 517,5820

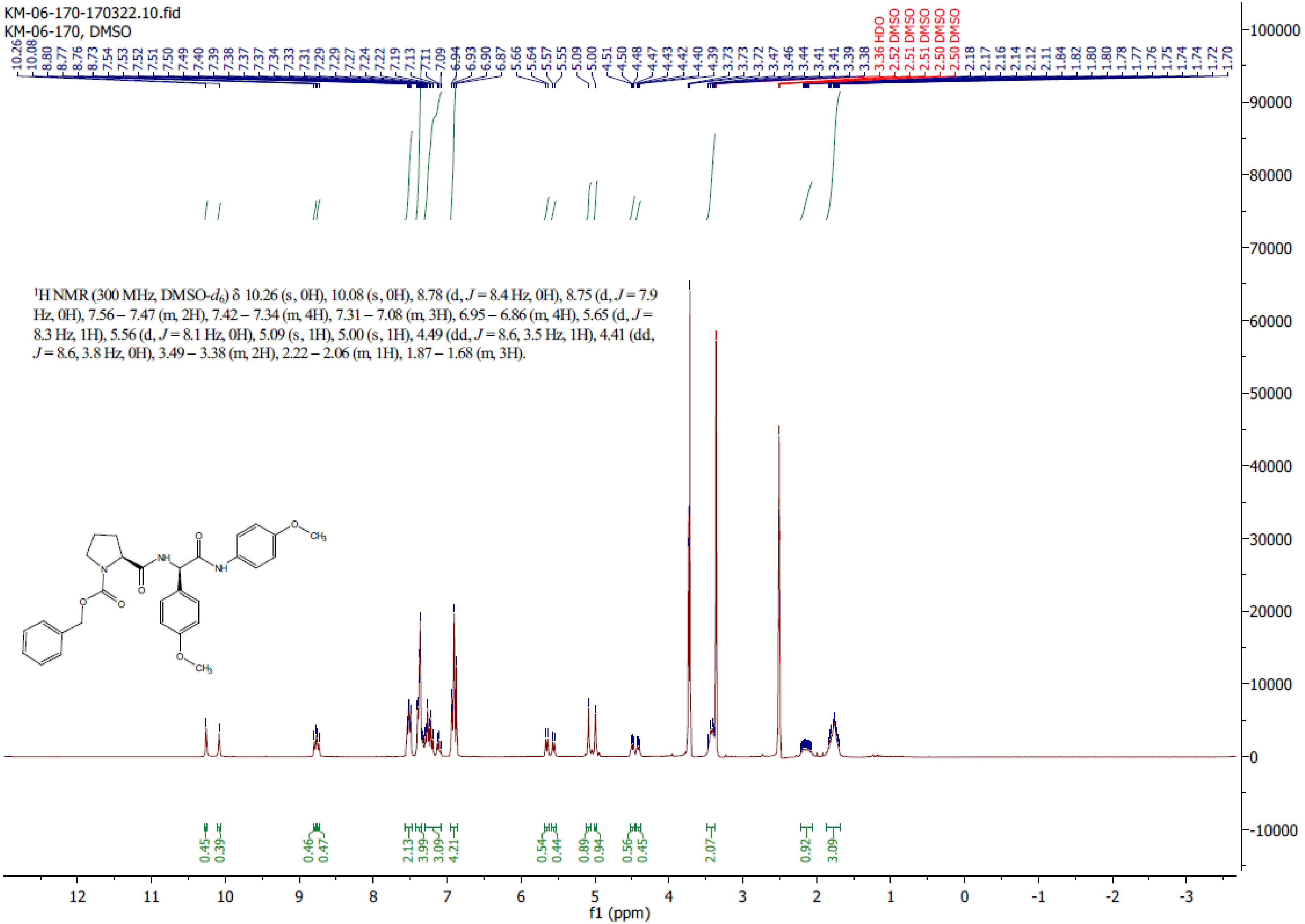
KM-06-170-170322.10.fid
KM-06-170, DMSO
^{1}H NMR (300 MHz, DMSO-d_6) δ 10.26 (s, 0H), 10.08 (s, 0H), 8.78 (d, J = 8.4 Hz, 0H), 8.75 (d, J = 7.9 Hz, 0H), 7.56 – 7.47 (m, 2H), 7.42 – 7.34 (m, 4H), 7.31 – 7.08 (m, 3H), 6.95 – 6.86 (m, 4H), 5.65 (d, J = 8.3 Hz, 1H), 5.56 (d, J = 8.1 Hz, 0H), 5.09 (s, 1H), 5.00 (s, 1H), 4.49 (dd, J = 8.6, 3.5 Hz, 1H), 4.41 (dd, J = 8.6, 3.8 Hz, 0H), 3.49 – 3.38 (m, 2H), 2.22 – 2.06 (m, 1H), 1.87 – 1.68 (m, 3H).
f1 (ppm)

KM-06-170-170322.11.fid
KM-06-170, DMSO

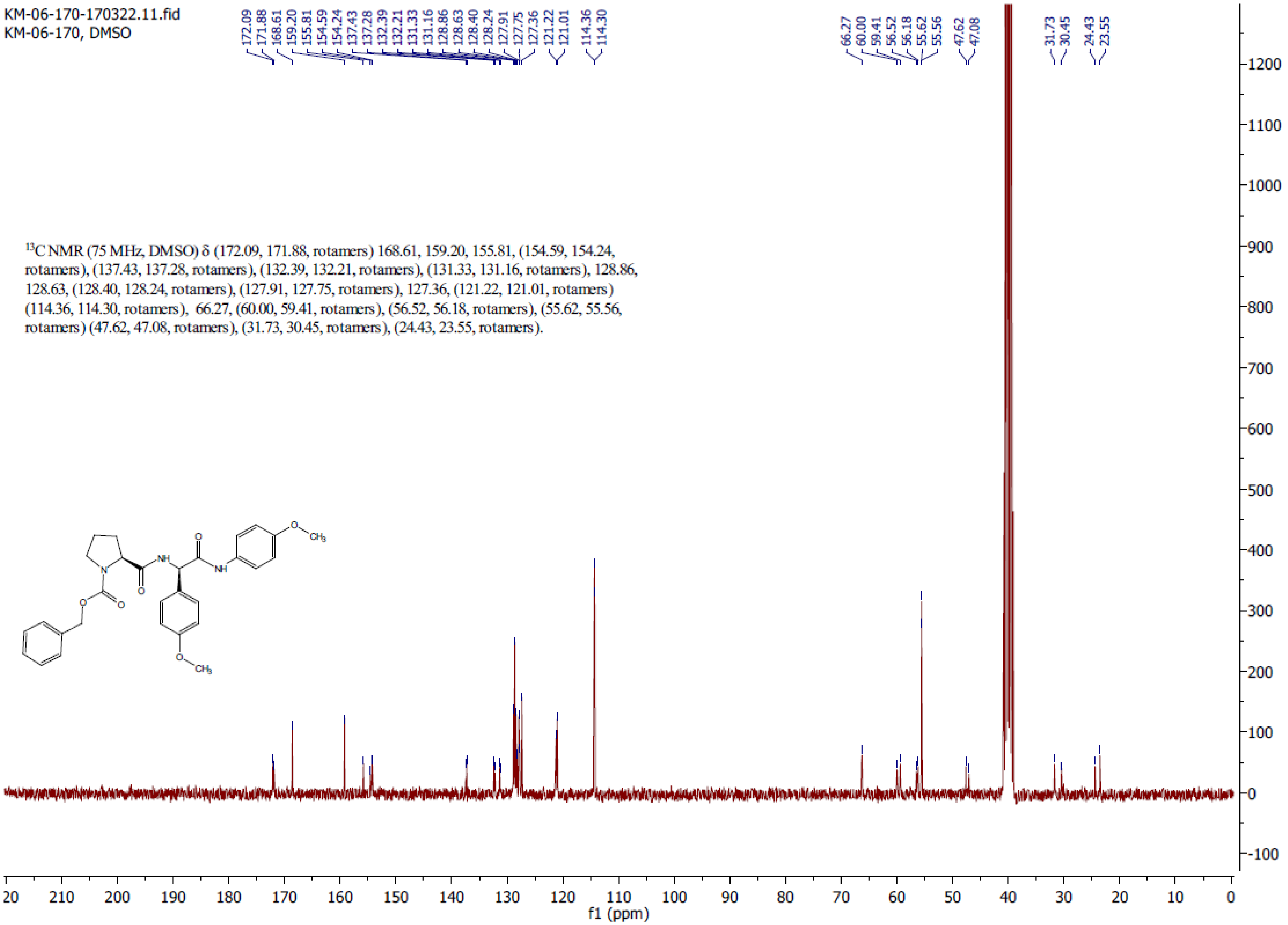

^{13}C NMR (75 MHz, DMSO) δ (172.09, 171.88, rotamers) 168.61, 159.20, 155.81, (154.59, 154.24, rotamers), (137.43, 137.28, rotamers), (132.39, 132.21, rotamers), (131.33, 131.16, rotamers), 128.86, 128.63, (128.40, 128.24, rotamers), (127.91, 127.75, rotamers), 127.36, (121.22, 121.01, rotamers) (114.36, 114.30, rotamers), 66.27, (60.00, 59.41, rotamers), (56.52, 56.18, rotamers), (55.62, 55.56, rotamers) (47.62, 47.08, rotamers), (31.73, 30.45, rotamers), (24.43, 23.55, rotamers).

Qualitative Analysis Report

User Chromatograms

Fragmentor Voltage 150 **Collision Energy** 0 **Ionization Mode** ESI

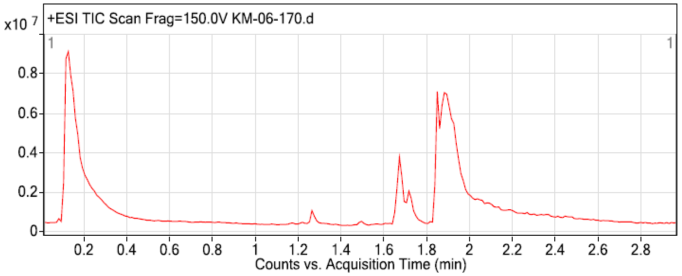

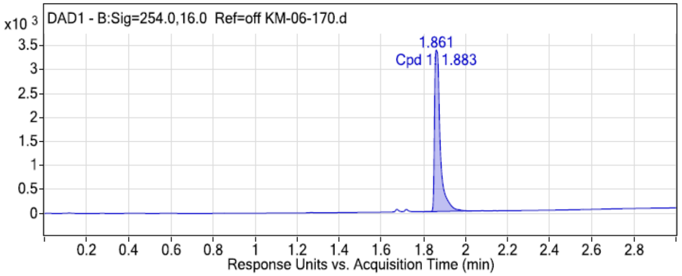

Integration Peak List

Peak	Start	RT	End	Height	Area	Area %
1	1.808	1.861	2.029	3362.88	6440.1	100

Compounds

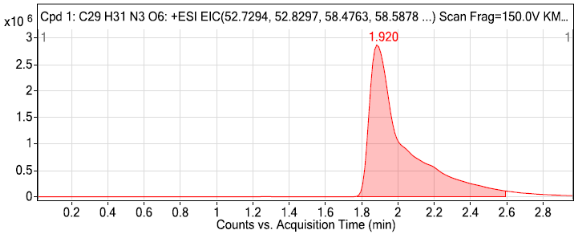

 Printed at: 12:44 AM on: 3/27/2017

Qualitative Analysis Report

User Spectra

Fragmentor Voltage	Collision Energy	Ionization Mode
150	0	ESI

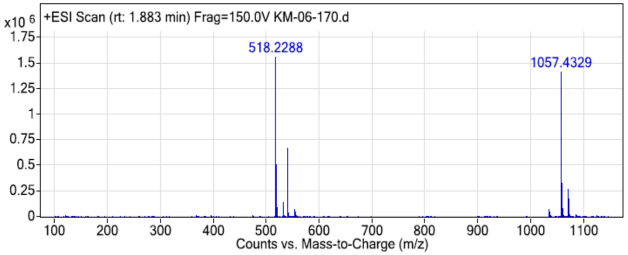

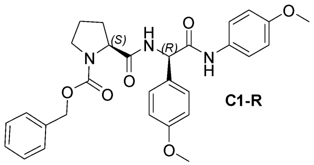

C1-R

Chemical Formula: $C_{29}H_{31}N_3O_6$
Exact Mass: 517,2213
Molecular Weight: 517,5820
