# Supplementary figures and images for: Small molecule inhibiting microglial nitric oxide release could become a potential treatment for neuroinflammation
Source: PLoS One. 2023 Feb 6;18(2):e0278325. doi: 10.1371/journal.pone.0278325 (PMC9901772; doi:10.1371/journal.pone.0278325)

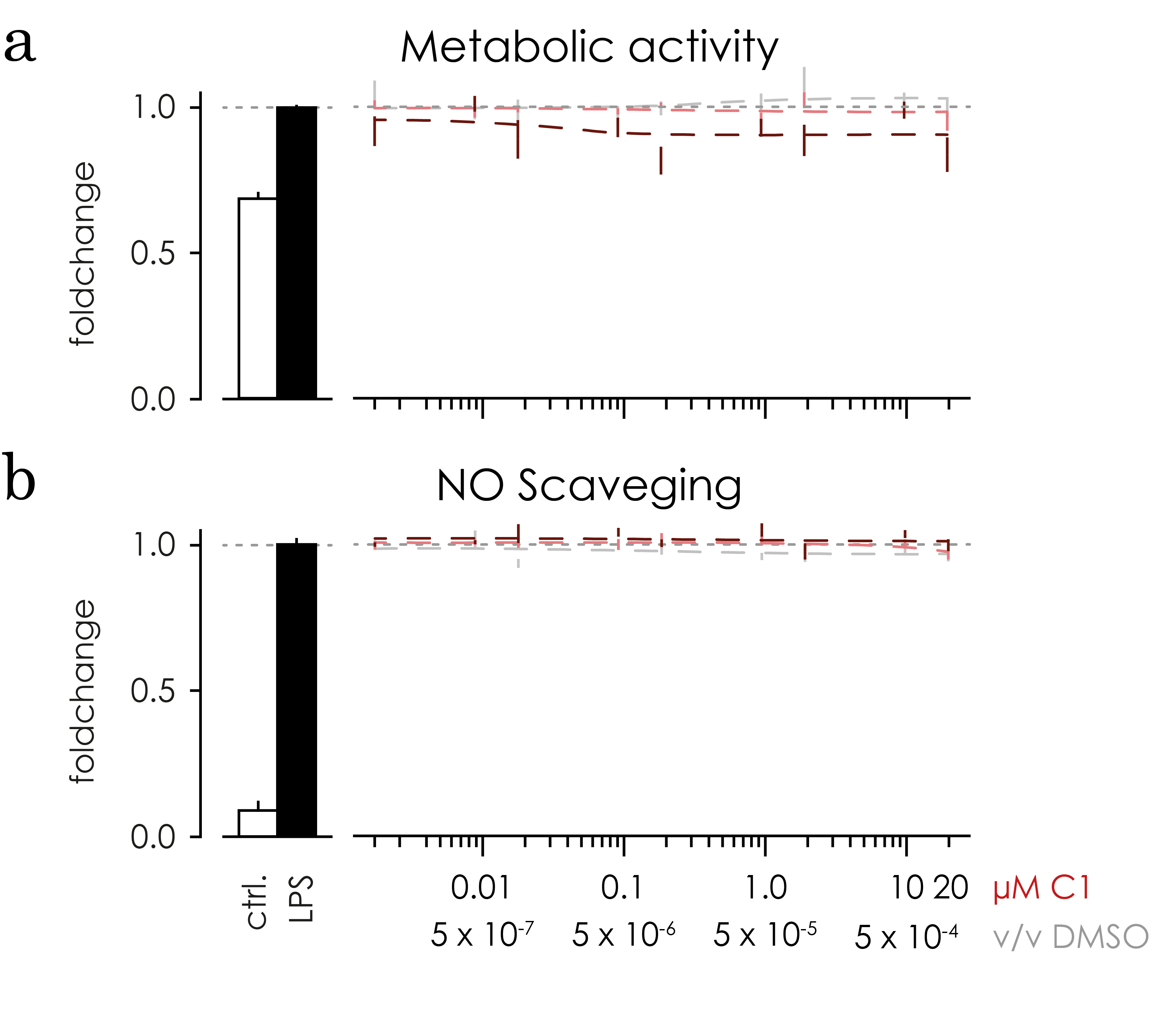

Supplement: S1 Fig — a) The metabolic activity of primary cultured neonatal microglia was measured using an AlamarBlue assay. DMSO alone showed no impact on the metabolic activity of the cells. 20 μM of C1 reduced the metabolic activity to 84% ± 3.99%, however not below the unstimulated control (69% ± 2.47%, white bar). b) C1 and DMSO do not scavenge NO or independently impact the Griess assay. NO enriched supernatant taken from LPS stimulated microglia (1 μg/mL LPS for 48 hours) was incubated with C1 in DMSO or DMSO alone (concentration range is the same as in a) for 24 hours. Treatment with C1 or DMSO showed no dose dependent reduction on the NO concentration in the supernatant when compared to the untreated supernatant. (PNG) [file pone.0278325.s003.png]

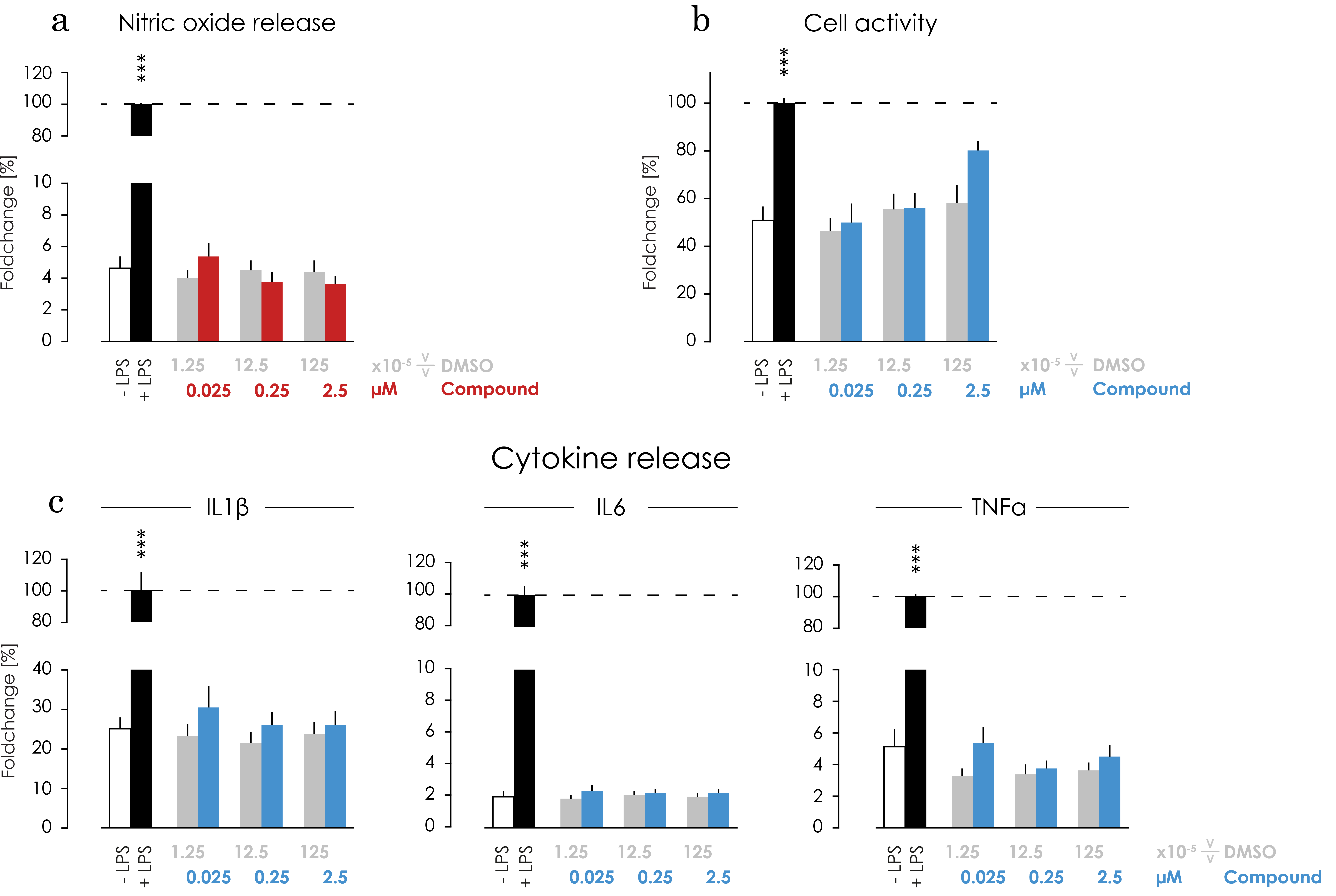

Supplement: S2 Fig — a) The effect of C1 on the NO release in unstimulated microglia showed no significant difference. Microglia were treated with C1 (0.025 μM, 0.25 μM, or 2.5 μM, in red) or its corresponding concentration of DMSO (1.25x10^-5^, 12.5x10-5, or 125x10-5 v/v, in grey) for 48 hours. Untreated microglia stimulated with 1 μg/mL LPS for 48 hours were set as positive control (in black). All values were normalised to the positive control. As shown in Fig 1, LPS stimulation induced a significant increase in NO release (p<0.0001). Treatment with C1 or DMSO did not show any significant difference compared to untreated microglia (in white). b) Using the same protocol as in a, the metabolic activity was assessed using AlamarBlue assay. LPS stimulation induced a significant increase in metabolic activity (in black) compared to the unstimulated microglia (in white) (p<0.0001). Treatment with C1 (in blue) or DMSO (in grey) did not show any significant difference compared to untreated microglia. c) Treatment with C1 (in blue) did not induce any significant changes in the release of pro-inflammatory cytokines (left: IL1β, middle: IL6, right: TNFα). Using the same protocol as described in Fig 2 the release of IL1β, IL6, and TNFα was measured using ELISA. Untreated microglia stimulated with 1 μg/mL LPS for 48 hours were set as positive control (in black). All values were normalised to the positive control respectively. As shown in Fig 2 LPS stimulation induced a significant increase in the release of IL1β, IL6, and TNFα (all p<0.0001). Treatment with C1 (in blue) or DMSO (in grey) did not show any significant difference compared to untreated microglia in all measured cytokines. *** p<0.001 comparing to plain medium control (1way ANOVA followed by Bonferroni’s post-hoc test). (PNG) [file pone.0278325.s004.png]

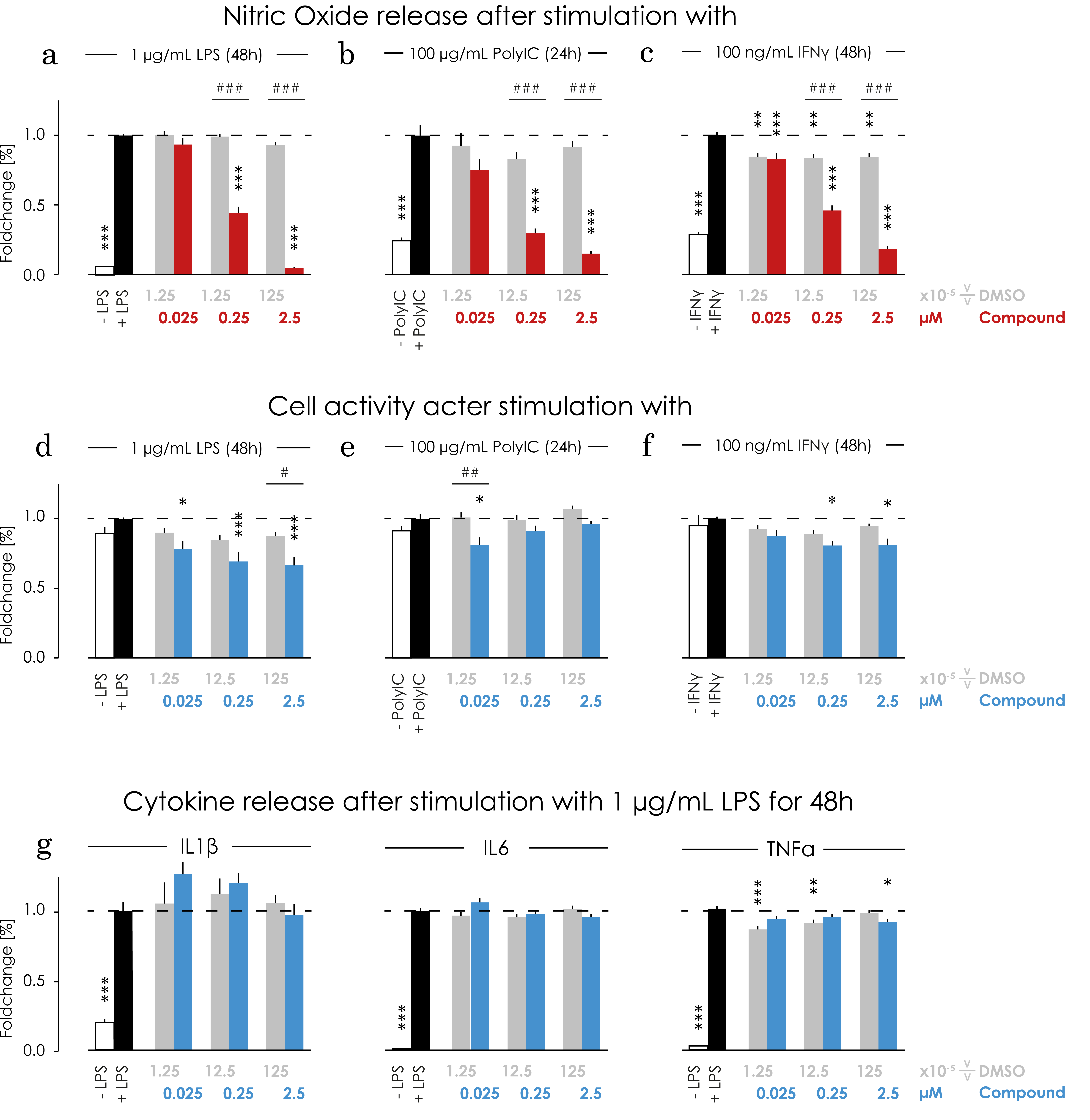

Supplement: S3 Fig — a) Adult bone marrow derived macrophages were isolated and pre-treated for 1 hour with C1 (0.025 μM, 0.25 μM, or 2.5 μM, in red) or its corresponding concentration of DMSO (1.25x10^-5^, 12.5x10-5, or 125x10-5 v/v, in grey), followed by 48 hours stimulation with 1 μg/mL LPS. In addition, untreated macrophages were stimulated 48 hours with 1 μg/mL LPS (in black) or kept in plain medium (negative control in white). 0.25 μM C1 reduced the NO release significantly compared to positive control (p < 0.0001) and DMSO (p < 0.0001) and 2.5 μM C1 reduced the NO release to similar level as the negative control (C1: 4.85% ± 0.65 SEM, plain medium: 6.01% ± 0.62 SEM, p > 0.9999) significantly different to the positive control (p < 0.0001) and DMSO (p < 0.0001). b) The same stimulation protocol as in a) was used, replacing the LPS by 100 μg/mL PolyIC for 24 hours and the NO release was measured. Similar as for the LPS stimulation, 0.25 μM showed a significant decrease in NO concentration compared to positive control (p < 0.0001) and DMSO (p < 0.0001). 2.5 μM of C1 lead to a decrease (15.01±10.32%, p = 0.9751) to the same NO concentration as induced by the negative control (24.30±12.91%), which is significantly different from the positive control (p < 0.0001) and DMSO (p < 0.0001). c) When the stimulus of the protocol, used in a, was exchanged by 100 ng/mL IFNγ (48 hours stimulation), C1 reduced the NO release in a dose dependent manner, reaching the same level as the negative control when using 2.5 μM C1 (control 29% versus treated 18%, p = 0.1253) and DMSO reduced the IFNγ induced NO release in these cells independently of the applied dose (1.25x10^-5^ v/v: 85%, p<0.0043; 12.5x10-5 v/v: 83%, p<0.0016; 125x10-5 v/v: 85%, p<0.0046), similar to the effect observed in microglia when stimulated with IFNγ. d) When stimulated with 1 μg/mL LPS (for 48 hours, using the same protocol as referred above C1 caused a dose independent decrease in cell activity for all 3 concentrations of C1 (0.025μM [file pone.0278325.s005.png]

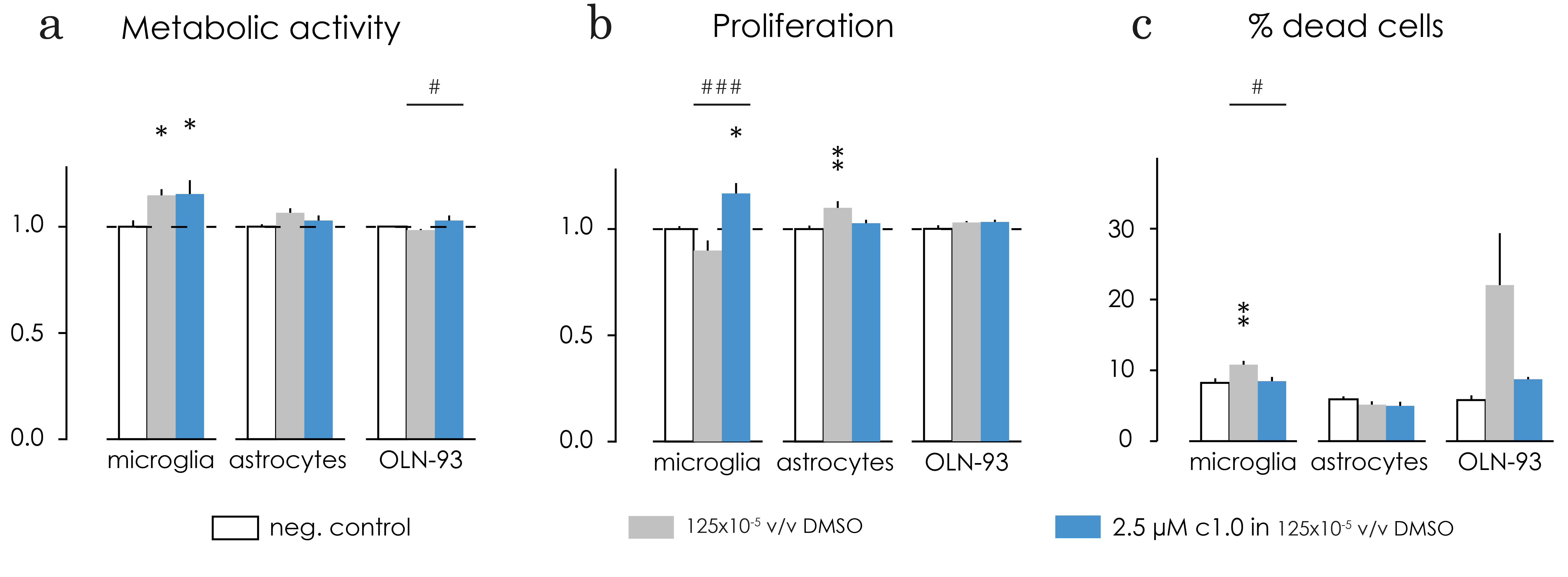

Supplement: S4 Fig — a) The metabolic activity of primary cultured neonatal microglia, primary cultured neonatal astrocytes and the oligodendrocyte cell line OLN-93 was evaluated under physiological condition (unstimulated). The cells were treated for 48 hours with 2.5 μM C1 (in blue), or its corresponding concentration of DMSO (125x10-5 v/v, in grey) or plain medium (in white). DMSO (p = 0.0439) and C1 (p = 0.0402) showed a significant increase in metabolic activity on microglia compared to plain medium. Comparing DMSO and C1 showed no significant difference. DMSO and C1 showed no influence on astrocytes. C1 increased the metabolic activity in oligodendrocytes compared to DMSO (p = 0.0431) but not compared to plain medium (p = 0.2140). b) Using the same experimental setup as in Fig 3, the proliferation and cell death was measured using a propidium iodide based assy. C1 increased the proliferation in microglia significantly compared to plain medium (p = 0.0154) and DMSO (p < 0.0001), but did not show an effect on the other tested cell types. DMSO did increase the proliferation of astrocytes compared to plain medium (p = 0.0060). c) DMSO increased the percentage of dead cells in microglia significantly compared to plain medium (p = 0.0096). Treatment with C1 kept the percentage of dead cells similar to plain medium (C1: 8.49%, plain medium: 8.26%) with no significant difference. However, it showed a significant reduction from the elevated DMSO level (p = 0.0201). ** p<0.01 * p<0.05 comparing to stimulated control, ### p<0.001 ## p<0.01 # p<0.05 compared to DMSO (1way ANOVA followed by Bonferroni’s post-hoc test). (PNG) [file pone.0278325.s006.png]
